# Advances in solid handling for continuous flow synthesis of specialty chemicals and pharmaceuticals

**DOI:** 10.1038/s42004-026-01954-3

**Published:** 2026-02-23

**Authors:** Zen Johnston, Thabo Peme, Tommy Mabasa, Christophe Len, Darren Riley, Jenny-Lee Panayides, Cloudius Ray Sagandira

**Affiliations:** 1 Department of Chemistry, University of Pretoria, Pretoria, South Africa; 2 Pharmaceutical Technologies, Council for Scientific and Industrial Research, Pretoria, South Africa; 3 Chimie ParisTech, CNRS, PSL Research University, Institute of Chemistry for Life and Health Sciences, Paris, France

**Keywords:** Synthetic chemistry methodology, Sustainability, Process chemistry, Flow chemistry, Chemical engineering

## Abstract

Continuous flow chemistry has transformed the synthesis of pharmaceuticals and specialty chemicals by advancing sustainability, efficiency, and process control. Despite these advantages, the management of solids remains a major challenge, often leading to clogging, inefficient mixing, and limitations in scalability. This review discusses recent strategies developed to overcome these obstacles, including the use of continuous stirred-tank reactors, packed-bed reactors with immobilized reagents, reaction design modifications, Pickering emulsions, colloidal nanoparticle suspensions, and specialised equipment such as agitated tubular reactors, spinning disk reactors, and sonicated systems. By critically assessing these developments, we chart the trajectory toward more resilient and robust flow-based manufacturing, consolidating continuous flow chemistry as a cornerstone of modern chemical manufacturing.

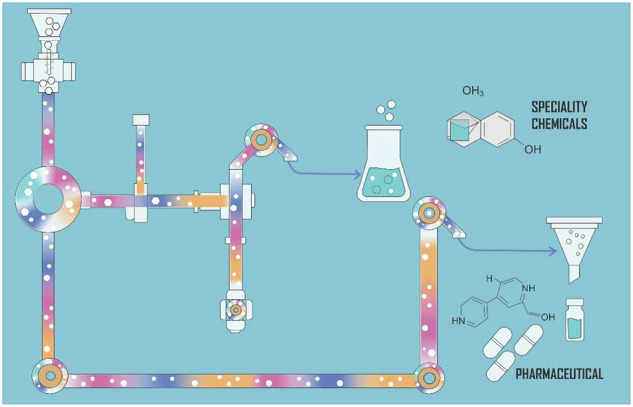

## Abstract (web summary)

Continuous flow chemistry enhances pharmaceutical and specialty chemical synthesis but struggles with solid management, causing clogging and scalability issues. Here, the authors review recent strategies developed to overcome these obstacles, highlighting advancements that strengthen flow-based manufacturing’s role in chemical production.

## Introduction

Continuous flow technology has garnered substantial interest in the specialty chemical and pharmaceutical industries over the past few decades, establishing itself as a transformative platform for process intensification^1–3^. This surge in adoption is driven by its alignment with societal imperatives for efficient, environmentally benign, and sustainable manufacturing processes, coupled with the escalating demand for novel chemical entities and pharmaceutical products^4–6^. The technology’s appeal stems from its ability to deliver a suite of operational advantages over traditional batch processing, including enhanced process safety, superior heat and mass transfer, accelerated reaction kinetics, precise control of reaction parameters, improved reproducibility and product quality, elevated yields and selectivity, rapid process scalability, reaction telescoping, automation, and in-line analytical and purification capabilities^4–12^. Moreover, continuous flow systems enable the execution of reactions under conditions previously unattainable in batch reactors, such as high-pressure, high-temperature, or highly reactive conditions, thereby expanding the synthetic toolkit available to chemists^13–16^.

Despite these benefits, a persistent challenge in the development and implementation of continuous flow processes is the effective management of solids, which poses significant operational hurdles, particularly in upstream processing^17–19^. The characteristic micro- or milli-scale reactor channels in continuous flow systems, while advantageous for heat and mass transfer, are prone to clogging and fouling when solids are present. Solids encountered in these systems can be broadly categorised into three types: (a) solid starting reagents, such as heterogeneous catalysts or insoluble bases; (b) solid products, including crystalline or precipitated target compounds; and (c) solid by-products, such as inorganic salts or polymeric residues formed during the reaction. The increasing adoption of end-to-end continuous manufacturing also underscores the importance of downstream solid handling, encompassing processes like product precipitation, crystallisation, and filtration, which are critical for achieving high-purity pharmaceutical and chemical products^20–23^.

Most continuous flow systems are inherently designed and optimised for homogeneous, solution-phase chemistry, where reactants, catalysts, and products remain fully dissolved. However, many industrially relevant transformations necessitate the use of solid reagents or result in the formation of insoluble species, complicating process design. Notably, key reactions in pharmaceutical and fine chemical synthesis, including Suzuki-Miyaura cross-coupling, palladium-catalysed Buchwald-Hartwig amination, hydrogenation, and various alkylations, acylations, and arylations, commonly employ organometallic reagents or halide-containing substrates that produce poorly soluble halide salts such as NaCl and KBr as by-products^24–29^. These salts, due to their low solubility in common organic solvents, precipitate within reactor channels, leading to blockages that disrupt flow and compromise process efficiency. Similarly, heterogeneous catalysts, such as supported palladium or nickel, are often employed to enhance reaction selectivity and recyclability but introduce additional solid handling challenges due to their physical presence in the reactor.

To mitigate these issues, researchers have pursued a multifaceted approach to solid handling in continuous flow systems, integrating advances in reactor engineering and process chemistry^18,29–35^. Reactor design innovations, such as wider channel diameters, segmented flow regimes, or specialised mixing zones, aim to minimise clogging while maintaining the benefits of microreactor technology^31,34,36,37^. Mechanical interventions, including the application of vibrational energy or ultrasonication, have been explored to dislodge or prevent solid accumulation within reactor channels. Ultrasonication leverages acoustic cavitation to disrupt particle aggregation and maintain suspension, thereby enhancing flow stability^18,31,32,34,38^. Process chemistry strategies, including solvent optimisation, co-solvent incorporation, temperature, reagent modification, and in situ solubilisation techniques, are also critical for managing solubility-limited systems. For instance, the use of polar aprotic solvents or specific additives can improve the solubility of inorganic by-products, while adjustments in temperature and pressure can be employed to control precipitation kinetics. Hayes and Mallia’s review on solid-handling strategies under continuous flow conditions primarily examined these approaches and was limited to managing solids formed during reactions^39^.

This comprehensive review highlights recent advances in upstream strategies for managing solids in continuous flow systems, supported by case studies that illustrate a range of solid-handling scenarios relevant to the synthesis of speciality chemicals and pharmaceuticals. These case studies encompass the deliberate use of solid reagents in line with green chemistry principles as well as strategies for handling solids formed during reactions, whether as desired products or by-products. The review also examines dynamic mixing techniques essential for maintaining uninterrupted flow and explores approaches for managing solid by-products to mitigate their impact on reactor performance and process continuity.

Rather than concentrating primarily on reactor engineering and design, this review emphasises process chemistry–driven strategies for solid handling, offering a comprehensive overview of practical solutions tailored to different solid types and reaction conditions. Through the analysis of real-world applications, it provides researchers with actionable guidance for selecting appropriate solid-handling approaches in specific continuous flow contexts. The review also identifies current methodological gaps and outlines opportunities for future innovation, including the development of integrated solid-handling platforms and advanced in-line monitoring tools capable of predicting and preventing clogging events. By addressing one of the key challenges limiting broader implementation, this work aims to advance the adoption of continuous flow technology in pharmaceutical and fine chemical manufacturing, thereby supporting the transition toward more sustainable and efficient production paradigms.

## Solid handling challenges in continuous flow

**Fig. 1:**
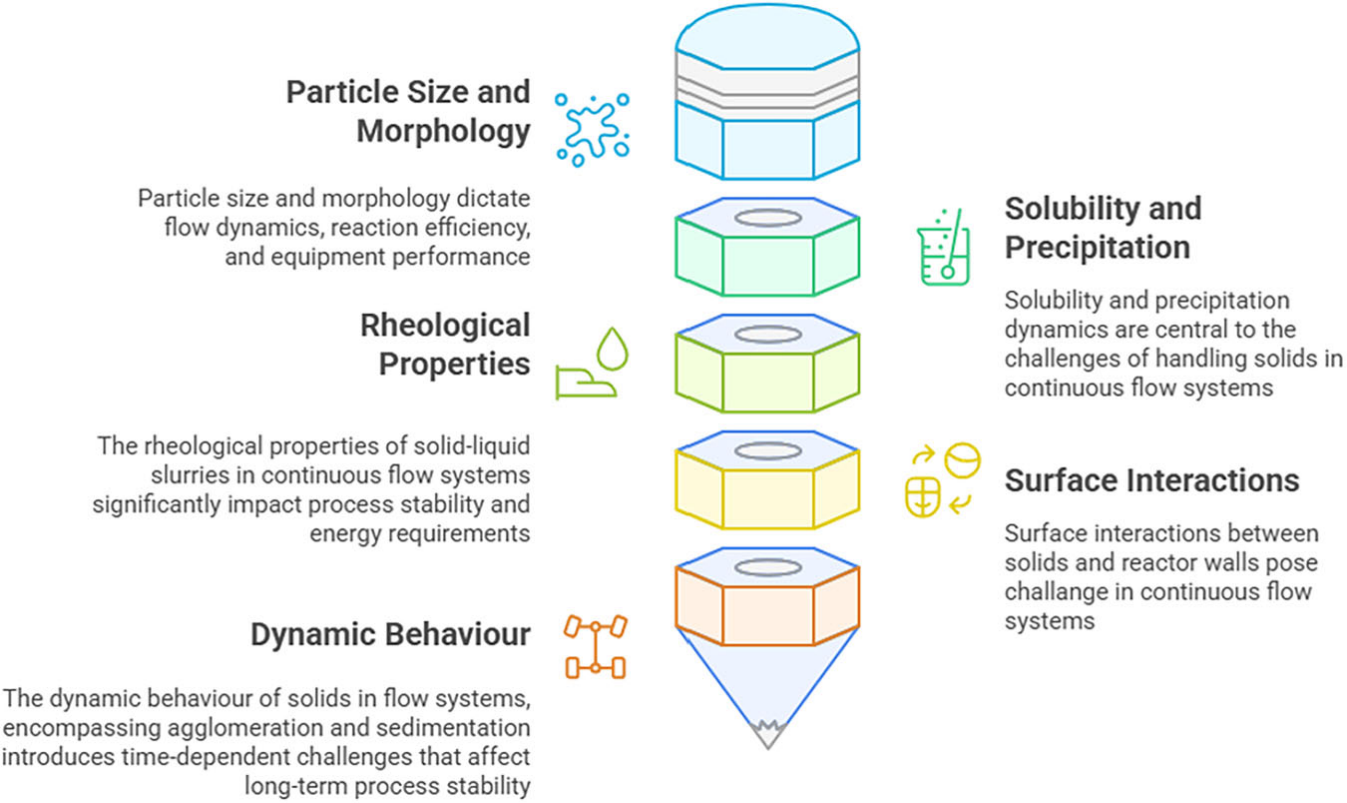
The nature and behaviours of solids in continuous flow systems. The effects of the nature and behaviours of solids in continuous flow systems separated into 5 categories.

The diverse nature and behaviours of solids in continuous flow systems create significant challenges that require careful consideration in process design, equipment selection, and operational strategies (Fig. 1). By understanding the specific properties of the solids being handled and employing tailored solutions, these challenges can be mitigated to achieve reliable, efficient, and safe continuous processing.

### Nature of solids and their behaviours

#### Particle size and morphology

The behaviour of solids in continuous flow systems is heavily influenced by particle size and morphology, which dictate flow dynamics, reaction efficiency, and equipment performance^39–41^. Particle size plays an important role in continuous-flow systems because it directly influences surface area, mass transfer, and the propensity for clogging. Fine particles, typically less than 10 μm, possess high surface area, which can enhance reaction rates in catalytic or reagent-driven processes^29,39^. However, their small size increases the likelihood of agglomeration due to van der Waals forces, leading to blockages in reactor frits or narrow channels^29,42^. This is particularly problematic in microreactors, where channel diameters are very small.

Conversely, larger particles (> 100 μm) reduce surface area per unit mass, lowering reaction efficiency in processes reliant on surface-mediated reactions, such as heterogeneous catalysis or adsorption^41,43^. Larger particles also settle more readily under gravity, leading to sedimentation in horizontal flow systems or low-velocity regions. This sedimentation can create uneven concentration profiles, reducing process uniformity.

Particle morphology further complicates flow behaviour. Spherical particles, often idealised in theoretical models, flow more predictably due to uniform drag and reduced interparticle friction^31,44^. However, real-world solids, such as needle-like crystals, exhibit anisotropic flow behaviour. Needle-like particles can interlock, increasing the risk of clogging, especially in constricted regions such as bends and valves. Irregular morphologies also increase wall friction, contributing to fouling.

#### Solubility and precipitation

Solubility and precipitation dynamics are central to the challenges of handling solids in continuous flow systems. Insoluble reagents, including heterogeneous catalysts, as well as insoluble by-products, such as halide salts generated in alkylations, acylations, and arylations reactions, pose significant risks of precipitation^25,41,42^. These solids can form aggregates that obstruct reactor channels, particularly in organic solvents with low dielectric constants, where ionic species have limited solubility. Solids may disperse into the liquid phase through three primary mechanisms: hydrodynamic bridging, settling, and fouling leading to eventual blockages of reactor channels^39^.

#### Rheological properties

The rheological properties of solid-liquid slurries in continuous flow systems significantly impact process stability and energy requirements. High solid loadings, to maximise throughput, increase slurry viscosity, leading to increased pressure drops across reactors^25,31,39,45,46^. For Newtonian fluids, viscosity is independent of shear rate, and pressure drop can be predicted using the Hagen-Poiseuille equation for laminar flow^39,45^. However, many slurries exhibit non-Newtonian behaviour, such as shear-thinning or shear-thickening properties^45,46^. As such, non-Newtonian behaviour complicates pumping and mixing. Flow instabilities, such as pulsations or channelling, arise when viscosity varies spatially due to segregation of solids or temperature gradients. These instabilities can lead to uneven residence times, reducing reaction uniformity.

#### Surface interactions

Surface interactions between solids and reactor walls, known as fouling, are a pervasive issue in continuous flow systems, particularly in microreactors with high surface-to-volume ratios^29,39^. Fouling occurs due to physical or chemical interactions, including electrostatic attraction, hydrophobic effects, or chemical bonding. For example, in aqueous systems, charged particles may adhere to oppositely charged reactor surfaces, while in organic solvents, hydrophobic solids like lignin or polymer residues can deposit on non-polar surfaces^31,39^. Fouling reduces effective channel diameters, increases pressure drops, and lowers heat transfer efficiency, compromising reactor performance.

In microreactors, where channel dimensions are small, even thin fouling layers can cause significant flow restrictions. Materials like stainless steel, commonly used in reactors, are prone to fouling due to surface roughness and chemical heterogeneity^31,39,45^. Polymeric reactors, such as those made from PTFE, may resist fouling by hydrophilic solids but are susceptible to hydrophobic deposits. Surface chemistry plays a critical role: for instance, silanol groups on glass reactors can form hydrogen bonds with polar solids, promoting adhesion

#### Dynamic behaviour

The dynamic behaviour of solids in flow systems, encompassing agglomeration and sedimentation, introduces time-dependent challenges that affect long-term process stability. Agglomeration occurs when particles collide and adhere, driven by van der Waals forces, hydrophobic interactions, or bridging by polymeric species^25,29,39,42^. In crystallisation processes, agglomeration of fine crystals can lead to large, irregular clusters that clog channels or alter product quality. Sedimentation, prevalent in low-velocity or horizontal flow systems, causes solids to settle, creating concentration gradients and reducing reaction efficiency^39,44,47^. These phenomena are particularly pronounced in processes with long residence times, where particles have more opportunities to interact.

### Fluid delivery

Pumps are fundamental components in continuous flow synthesis, serving as the driving force for the precise and controlled movement of reagents, solvents, and products through reactors and associated equipment^39,48,49^. Their role is critical to maintaining the steady-state operation, reproducibility, and efficiency that define continuous flow systems, distinguishing them from traditional batch processes. Introducing solid reagents into continuous flow systems presents distinct challenges, as conventional pumps are often not suited to handle particulate matter. The limitations of standard fluid delivery devices, combined with the unique properties of solids, necessitate specialised equipment and careful process design.

Delivering solid reagents, such as powders, granules, or slurries, into continuous flow systems is inherently complex due to the physical constraints of standard pumping technologies^29,31,39,47^. Unlike liquids or gases, solids can settle, agglomerate, or clog narrow passages, making it difficult to achieve consistent and reliable delivery. Slurries add further complexity, requiring continuous mixing in the reservoir and, critically, uniform uptake throughout the process. The choice of fluid delivery device significantly impacts process efficiency, equipment longevity, and the ability to maintain continuous operation in chemical synthesis and pharmaceutical manufacturing. The following sub-sections evaluate the major pump types used in flow chemistry, highlighting their strengths and limitations to guide the effective handling of solids in continuous flow synthesis.

#### Piston and syringe pumps

**Fig. 2:**
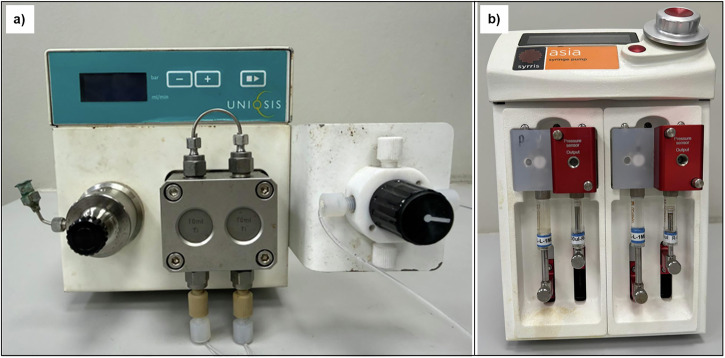
Syringe and piston pumps commonly utilised for continuous flow chemistry. **a** Photograph of a typical piston pump and **b** Photograph of a typical syringe pump.

These pumps are designed for precise delivery of homogeneous liquids and are widely used in continuous flow systems for their accuracy and ability to handle high pressures, often exceeding 100 bar^49^. However, their narrow fluid paths, typically involving small-diameter tubing and valves, are prone to blockages when solids are present. In addition to clogging, sedimentation within the syringe barrel or pump chamber poses a major limitation when handling suspensions, as particles can settle during operation or between dosing cycles^49^. This settling disrupts flow consistency, causes concentration gradients, and may lead to incomplete delivery or pump malfunction^49^. Consequently, piston and syringe pumps are restricted to fully dissolved solutions or suspensions with negligible solid content (Fig. 2).

#### Peristaltic pumps

**Fig. 3:**
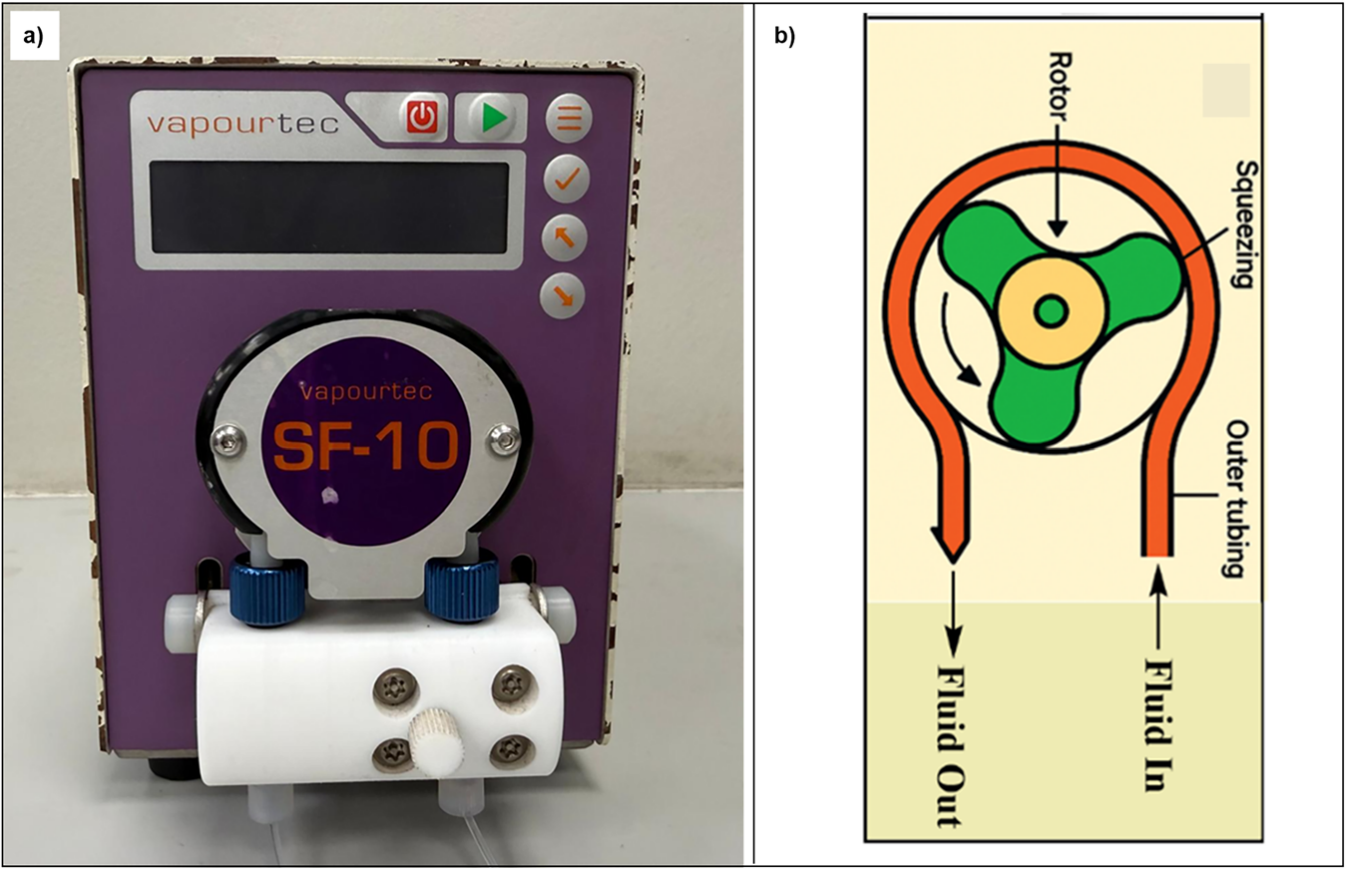
Typical peristaltic pump. **a** Photograph of a Vapourtec SF-10 standalone complete peristaltic pump. **b** Basic schematic displaying the inner functionality of a peristaltic pump with the inner rotor squeezing the tubing through which the fluid stream flows.

Peristaltic pumps offer a more viable solution for delivering solids, particularly in the form of either slurries or suspensions. These pumps operate by using a rotor to compress flexible tubing, commonly made from fluoropolymers like PTFE or FEP, creating a series of occlusions that propel the fluid forward (Fig. 3)^49^. For peristaltic pumps to function effectively, the particle size of solids must be smaller than the tubing diameter typically no more than one-third of that diameter and uniformly distributed to prevent settling or clogging^21,50^. Large or irregularly shaped particles can cause blockages or damage the tubing, necessitating pre-processing steps like grinding or sieving.

Peristaltic pumps have several advantages for handling solids, provided the particle size is carefully managed to avoid sedimentation or tubing blockages. The fluid remains confined within the tubing, preventing contact with the pump’s mechanical components, such as the rotor^21,50,51^. This reduces the risk of fouling, corrosion, or wear caused by abrasive or chemically reactive solids. The flexible tubing can accommodate some degree of solid content, but maintaining a stable suspension is critical to ensure consistent delivery. Solids that settle or agglomerate during pumping can lead to uneven flow rates or blockages, requiring additives such as surfactants or continuous agitation to keep particles dispersed^39^.

However, peristaltic pumps have a lower pressure tolerance, typically rated below 10 bar, which restricts their use in high-pressure reactions, such as catalytic hydrogenation^21,49–51^. Additionally, the pulsatile flow generated by the rotor can lead to inconsistent delivery rates, which may affect reaction uniformity in precision-driven applications. The tubing material must also be compatible with the chemical properties of the slurry and withstand repeated compression without degrading.

#### Diaphragm pumps

**Fig. 4:**
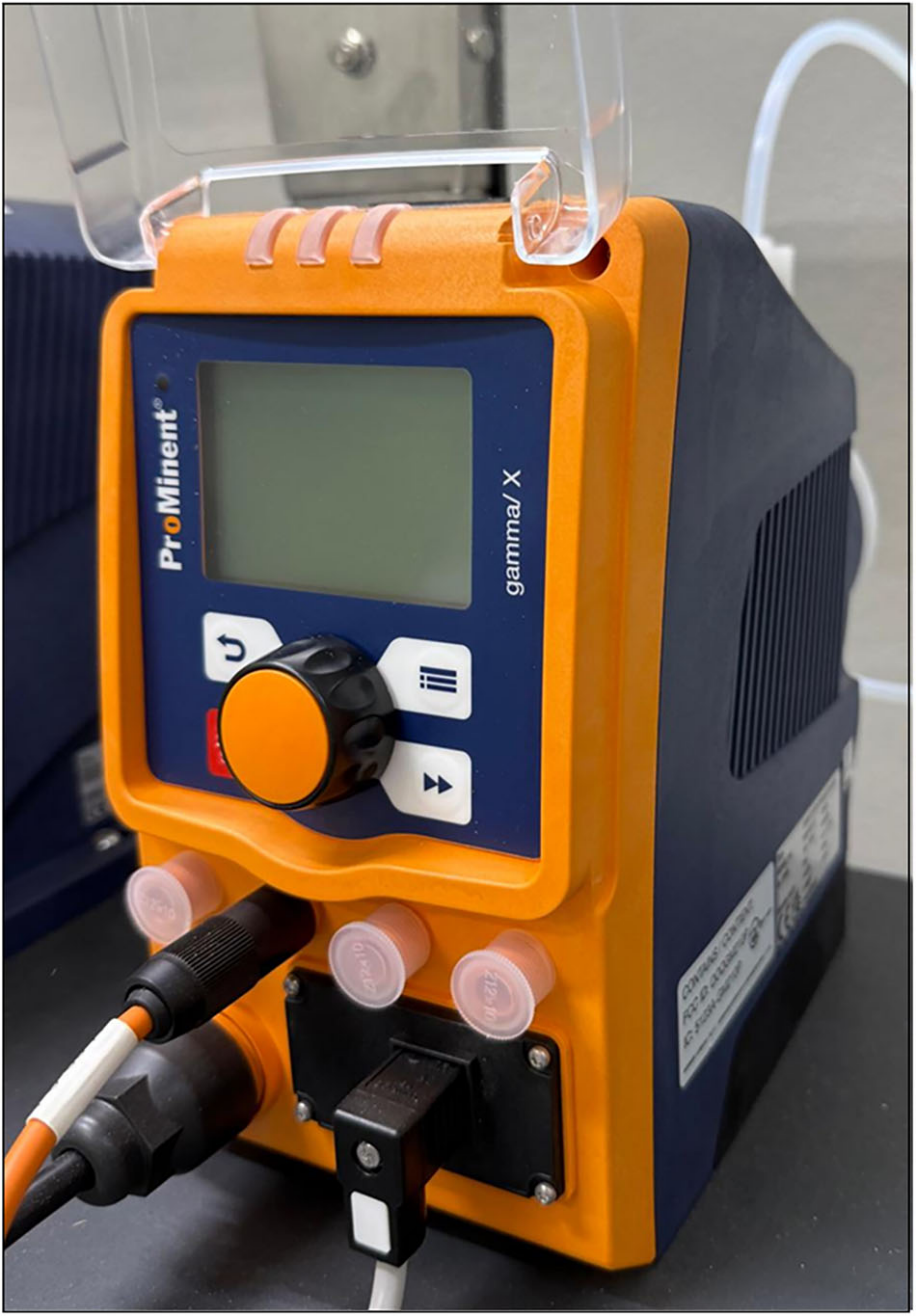
Typical diaphragm pump. Photograph of air-operated double diaphragm pump.

Diaphragm pumps are widely used in continuous flow synthesis to manage diverse fluids, including suspensions (1–20% solids by volume, low to moderate viscosity) and slurries (20–60% solids by volume, high viscosity) (Fig. 4). These pumps deliver consistent fluid volumes per stroke, independent of system pressure within operational limits^52^. Two primary variants, solenoid-driven diaphragm (SDD) pumps and air-operated double diaphragm (AODD) pumps, differ in their mechanisms and applications^52^.

SDD pumps utilise an electromagnetic solenoid to actuate a single diaphragm, achieving precise dosing (0.36–80 L.h^−1^; pressures up to 25 bar) with ±1% reproducibility, making them ideal for micro-dosing suspensions in laboratory-scale synthesis^52,53^. Conversely, AODD pumps employ compressed air to drive dual diaphragms, enabling high flow rates (1–1040 L.min⁻¹) and pressures up to 8.6 bar, suitable for industrial-scale processing of viscous slurries and solids up to 10 mm^52,54,55^.

Both pump types face challenges, including (i) pulsating flow, which can disrupt steady flow in continuous synthesis and is mitigated by pulsation dampers or multi-diaphragms, and (ii) diaphragm wear from abrasive or corrosive fluids, requiring periodic replacement^52,54^. However, the appropriate selection of materials tailored to the specific application significantly improves the durability of diaphragm pumps. SDD pumps are optimal for precise, low-flow applications, while AODD pumps excel in robust, high-throughput slurry handling, with pump selection dictated by fluid properties, flow rate, and pressure requirements.

Overall, introducing solid reagents into continuous flow requires careful alignment of pump capability with the physical behaviour of the solid–fluid mixture. Because particle size, viscosity, sedimentation, and pressure tolerance each impose specific constraints, the choice of delivery system is central to ensuring reliable, blockage-free operation. The following case studies and strategies illustrate how these principles play out in practice, linking various solid-handling approaches to operating windows, failure modes, and scale-up considerations, with key insights summarised in a comparative table.

## Solid handling approaches within continuous flow systems

In continuous flow synthesis, managing solids is critical to ensure process efficiency and prevent operational challenges such as clogging, uneven flow and steady state disruption. Solids in these systems manifest in three primary scenarios: (a) solid starting reagents, including heterogeneous catalysts (e.g., palladium on carbon), insoluble base (e.g., sodium carbonate) and immobilised regents (e.g., reagents bound on ion exchange resins); (b) solid products, such as crystalline and precipitated target compounds; and (c) solid by-products, such as either inorganic salts or polymeric residues formed during reactions. Each scenario necessitates tailored, solid handling strategies to maintain consistent flow and reaction efficiency.

This section reviews recent literature case studies to elucidate these approaches and evaluates their effectiveness in addressing the unique challenges posed by each solid type in continuous flow systems.

To provide a systematic and comprehensive discussion of solids-handling strategies in continuous processing, we classify the approaches according to their underlying mechanisms. These mechanisms encompass immobilisation through packed bed systems, dynamic mixing achieved with continuous stirred tank reactor cascades, spinning disc reactors, agitated tubular reactors, and oscillatory flow reactors or coiled oscillatory baffled reactors. Additional strategies include interfacial stabilisation using Pickering emulsions, nanoscale mobility through colloidal suspensions or simulated moving bed reactors, and process chemistry modifications involving the selection of bases, solvents, or alternative synthetic routes. This framework enables a clear comparison of how each approach addresses the inherent challenges posed by solids in continuous manufacturing environments.

For each category, we critically examine the operating windows that define practical applicability, summarise the advantages that make these strategies attractive, and identify potential risks and limitations. Scale-up considerations are discussed to bridge laboratory feasibility with industrial implementation, highlighting factors that influence robustness and reproducibility. The insights derived from this analysis are synthesised into a comparative table presented at the end of the section, providing a concise reference for rapid and informed decision-making. By organising these strategies within a unified framework, this review aims to guide researchers and practitioners in selecting solids-handling solutions that align with performance, sustainability, and scalability objectives in modern chemical manufacturing.

### Packed-bed reactors

Packed-bed reactors, also known as fixed-bed reactors, stand as a cornerstone in modern chemical engineering, offering a robust and adaptable solid-handling technique tailored to address the intricate demands of extreme solubility challenges^56,57^. These reactors are widely employed in applications including heterogeneous catalysis, scavenger systems, immobilised reagents, and inline purification, making them indispensable in both industrial and research settings^56–58^. Packed bed reactors are ideally suited for processes where solids have limited mobility or where immobilisation effectively reduces fouling risks. By anchoring catalysts or reagents onto fixed supports, these systems prevent the agglomeration and channel blockages commonly associated with fine particles^57^. Immobilisation also minimises sedimentation and ensures uniform contact between phases, thereby improving mass transfer and maintaining consistent flow conditions throughout the reactor. Packed-bed reactors minimise agglomeration and wall adhesion by constraining particle motion, reducing anisotropic bridging risks, and maintaining solids in a fixed position. This configuration enables precise fluid delivery using high-accuracy pumps, ensuring stable operation under solids-compatible conditions.

**Fig. 5:**
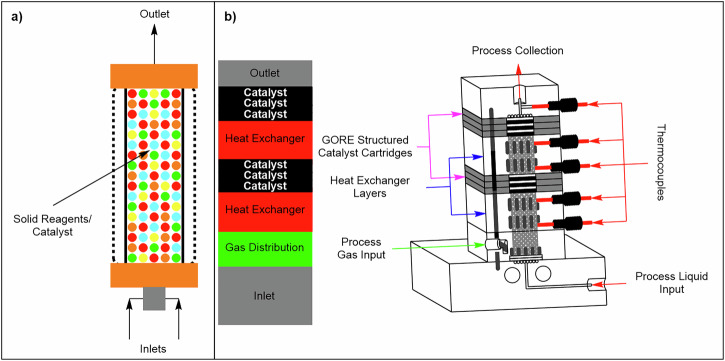
Two types of continuous flow packed-bed reactors. **a** Typical column packed-bed reactor. **b** Modular continuous flow packed-bed reactors with horizontal catalyst modules.

At their core, packed-bed reactors feature a housing vessel designed to securely immobilise solid reagents or catalysts, through which liquid and gaseous reagents are pumped, facilitating intimate contact and driving the reaction^49,59–61^. This interaction, predominantly governed by the surface area between the solid and liquid or gaseous phases, occurs within the reactor, where efficiency and yield depend on the precision of this interfacial contact. Packed-bed column reactors, a commonly used type of packed-bed reactors in continuous flow systems, are designed as hollow columns with inner cartridges made of glass, polymers, or stainless steel, housing packed solid reagents or catalysts (Fig. 5a)^49^. While packed-bed reactors are widely used at very large scales in processes such as refinery hydrotreating, hydrogenation, and desulfurization, their application in fine-chemical continuous flow presents distinct challenges^57^. These include channelling, uneven residence time distribution, high pressure drop, fouling, and catalyst attrition, which can compromise process control and reproducibility, especially with solids or viscous streams^58^. Numerous chemical transformations have been demonstrated at the laboratory scale using these reactors, but reports of successful scale-up in pharmaceutical manufacturing remain relatively scarce. To overcome these limitations, strategies such as structured packing, segmented flow, or dynamic mixing are often employed to maintain consistent performance.

Recently, Breen et al.^58^, reported a novel method for heterogeneous catalysis, addressing challenges in packed column-bed reactors with powdered catalysts by embedding them in a poly(tetrafluoroethylene) (ePTFE) matrix, enhancing handling ease. The modular design allows reconfiguration, ensuring uniform temperature, mixing, and low-pressure drop, facilitating seamless scale-up (Fig. 5b).

Packed-bed reactors offer many advantages, including efficient heterogeneous catalysis, uniform temperature control, enhanced mixing, and easy downstream processing. In this subsection, we highlight a selection of case studies on the use of packed-bed reactors in continuous flow synthesis, exploring how these methods overcome solid handling limitations.

**Fig. 6:**
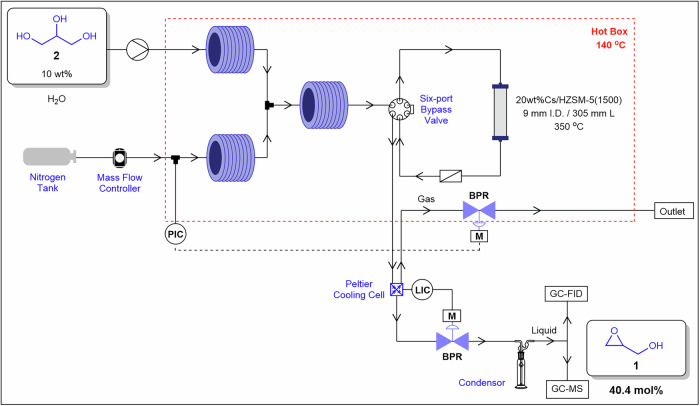
Continuous flow process for the preparation of **2**. Continuous flow scheme for the preparation of **1** from **2** with the use of a packed-bed reactor, reproduced from Kostyniuk and co-workers^62^.

Kostyniuk and co-workers^62^ demonstrated a novel, green and one-step continuous flow synthesis of glycidol **1** via gas-phase epoxidation of glycerol **2**, utilising a heterogeneous Cs-ZSM-5 zeolite catalyst in a packed-bed reactor (Fig. 6). Their approach differs from that of previously reported procedures utilising liquid-phase synthesis using either Brönsted or Lewis acids^63–66^. The authors achieved a highest yield of 40.4 mol% of **1** using a 20 wt% Cs–ZSM-5(1500) catalyst, with 10 wt% **2** in the feed, at 350 °C and a (gas hourly space velocity) GHSV_total_ of 1250 h⁻¹. The selectivity of **1** increased steadily from 41.4 to 64.3 mol% over 27 h of time-on-stream. The catalyst exhibited high stability and selectivity, attributed to an optimal concentration of basic sites and a synergistic interaction between CsNO₃ and the HZSM-5 support. The application of a column packed-bed reactor enabled excellent system performance under continuous flow conditions. This case highlights how immobilisation stabilises high-surface-area powders while reducing channel fouling and pressure buildup caused by wall interactions. Delivering reactants in the gas phase further avoids the viscosity challenges associated with slurries, ensuring efficient and reliable operation under solids-compatible conditions.

**Fig. 7:**
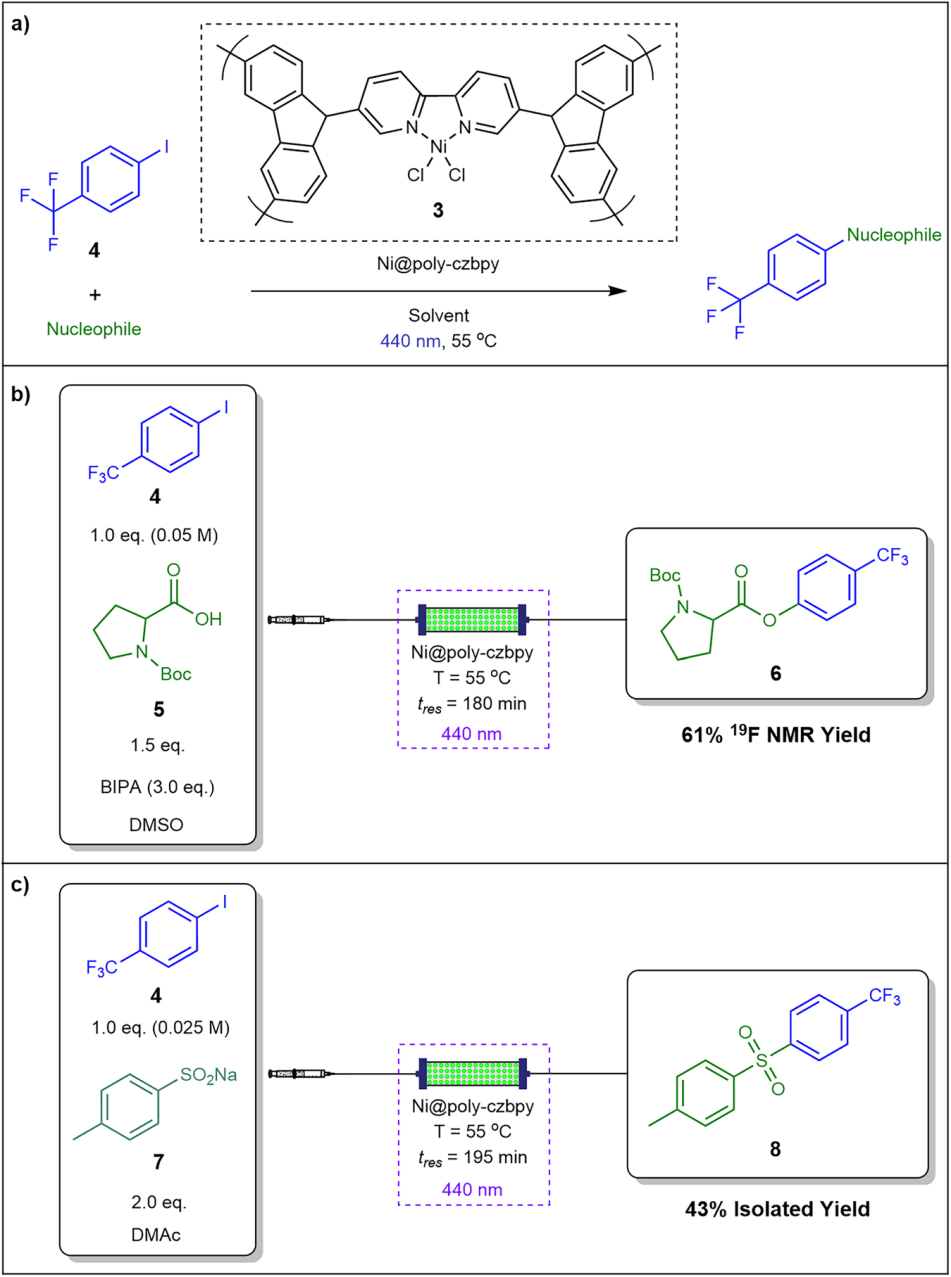
Heterogeneous catalysed C–O and C–S coupling in continuous flow. **a** General C–O and C–S coupling using catalyst **3**. **b** Packed-bed continuous flow system for the C–O coupling. **c** Packed-bed continuous flow system for the C–S coupling^67^.

Hsu et al.^67^ reported heterogeneous metallaphotoredox catalytic cross-coupling reactions in continuous flow. The authors utilised a poly-5,5’-di(9*H*carbazol-9-yl)-2,2’-bipyridine (poly-czbpy) **3** packed-bed reactor in which the nucleophile was reacted with 4-iodobenzotrifluoride **4** affording iodophenyl derivatives (Fig. 7a). Catalyst **3** was mixed with glass beads and silica for packing in the reactor. Initially, the authors demonstrated C–O coupling of 4-iodobenzotrifluoride **4** and *N*-(Boc)-proline **5** in the presence of *N*-*tert*-butylisopropylamine (BIPA) to afford **6** in 61% ^19^F-NMR yield (Fig. 7b). With the optimum conditions, **7** was successfully converted into **8** with 50% ^19^F-NMR yield and 43% isolated yield at 55 °C in 3 h total residence time (Fig. 7c). By immobilising the photocatalyst within a mixed-bead bed, this approach mitigates non-Newtonian slurry behaviour, prevents particle interlocking caused by irregular morphologies, and minimises fouling on transparent surfaces essential for maintaining photon flux.

**Fig. 8:**
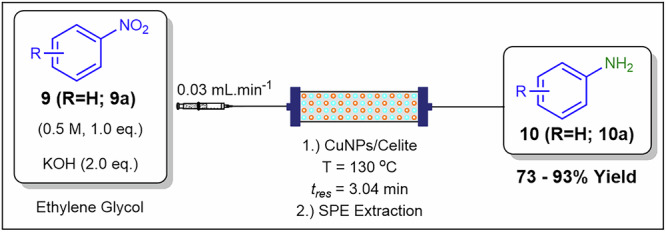
Continuous flow process for the preparation of **10**. Continuous flow for the reduction of nitrobenzene derivatives **9** to corresponding anilines **10** using CuNPs/Celite packed-bed reactor^68^.

Nanoparticle catalysts have emerged as highly effective tools in synthetic chemistry, primarily due to their significantly increased surface area, which enhances catalytic activity and reaction efficiency. The high surface-to-volume ratio of nanoparticles facilitates greater interaction between the catalyst and reactants, enabling faster reaction rates and improved selectivity. Martina and co-workers^68^ demonstrated the effectiveness of a packed-bed reactor system containing copper nanoparticles in reducing nitroarenes **9** to anilines **10** under continuous flow conditions (Fig. 8). The reduction of nitrobenzene **9a** to aniline **10a** was employed as a model reaction to optimise key parameters, including catalyst supports, solvents, and residence time. Optimal conditions were determined to be 0.5 M **9a**, KOH (2 eq.) and a 3.04 min residence time using a CuNPs/Celite packed-bed reactor (500 mg, 5% w/w) maintained at 130 °C, achieving quantitative conversions and a 93% isolated yield. Additionally, the authors conducted substrate scope studies with various nitroarenes to assess the method’s generality, yielding anilines in 73–93% yield. While nanoparticle dimensions enhance mass transfer, they often increase fouling susceptibility. Immobilising these particles on celite mitigates adhesion and enables liquid delivery via piston or syringe pumps without the instability associated with slurries.

Hydrogenation is a key transformation in chemical synthesis and is widely employed across industrial and academic settings to facilitate a range of critical reactions^57,58,69,70^. This process is predominantly achieved using heterogeneous catalysts, such as palladium on carbon (Pd/C) and Raney nickel, which offer robust and reusable platforms for catalytic activity^57,69^. Hydrogenation enables diverse transformations, including the reduction of nitro groups to amines, the saturation of alkenes, the conversion of carbonyl compounds to alcohols, and selective debenzylation and dehalogenation reactions^69–71^. These reactions are essential in the synthesis of pharmaceuticals, fine chemicals, and advanced materials, underscoring the versatility and importance of hydrogenation in modern chemistry. While continuous flow technology in hydrogenation improves safety, efficiency, and sustainability by addressing gas/liquid/solid multiphase mixing and the challenges of handling flammable H₂ (g) compared to batch processes, the management of solid catalysts remains a critical consideration.

**Fig. 9:**
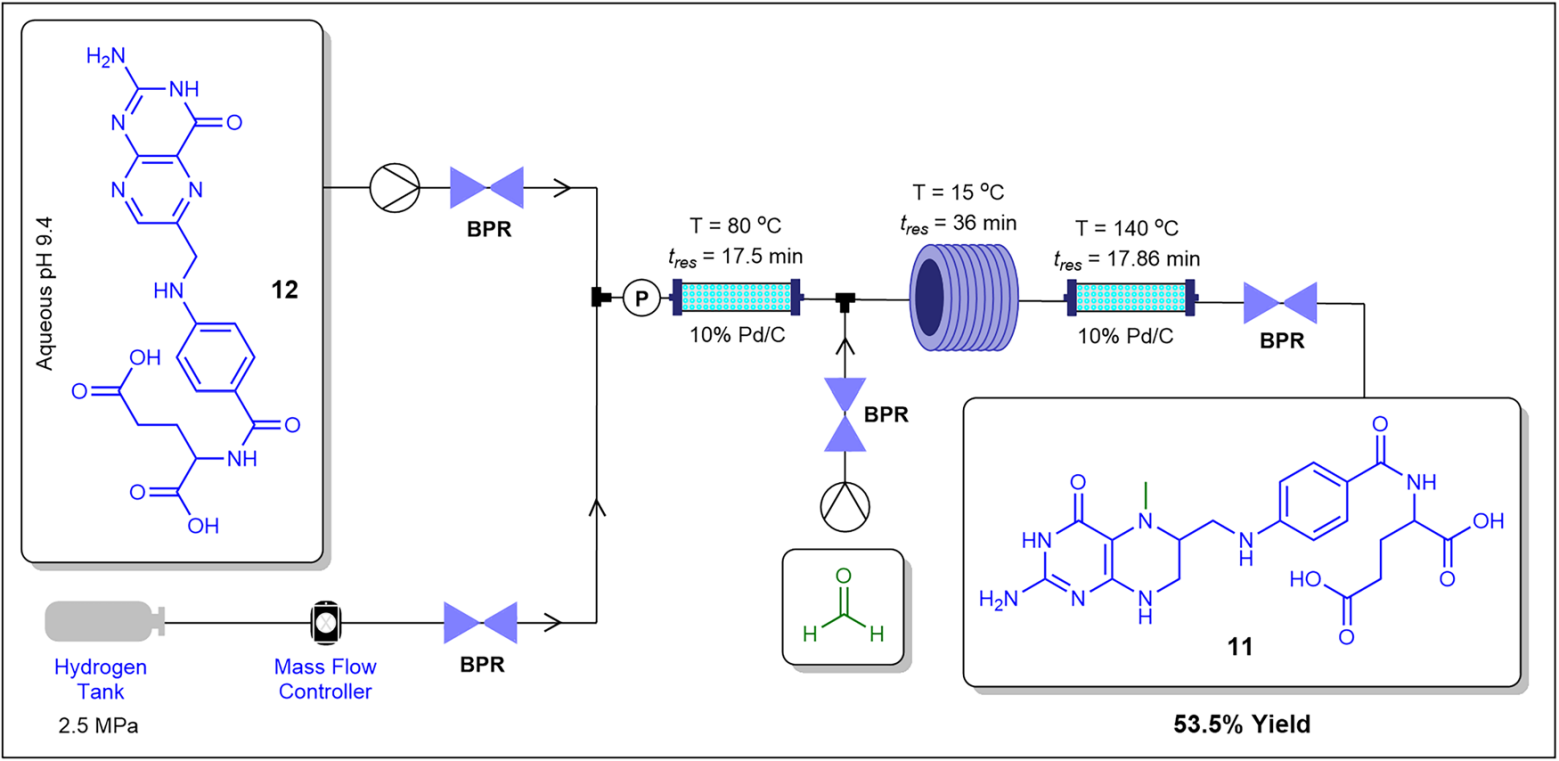
Continuous flow process for the preparation of **11**. Telescoped continuous flow synthesis of **11** from **12** via hydrogenation using Pd/C packed-bed column reactors.

Zhang et al.^72^ demonstrated a 3-step telescoped continuous flow synthesis of 5-methyltetrahydrofolate **11** from folic acid **12** (Fig. 9). The first step involved the hydrogenation of **12** to tetrahydrofolic acid in situ in a 10% Pd/C packed-bed column reactor held at 80 °C, followed by methylenation of tetrahydrofolic acid intermediate in a coil reactor at 15 °C to afford 5-ammonium methylenetetrahydrofolate and 5,10-methylenetetrahydrofolate. The products were subsequently hydrogenated in a 10% Pd/C packed-bed reactor held at 140 °C affording **11** in 53.5% overall yield over 71.36 min total residence time. Numerous hydrogenation case studies on the use of packed-bed column reactors under continuous flow conditions have been reported in the literature^57,69,70^. Fixed Pd/C beds decouple catalyst morphology from hydrodynamic effects, suppress precipitation-induced fouling across stages, and maintain stable pressure profiles compatible with precision pumping.

**Fig. 10:**
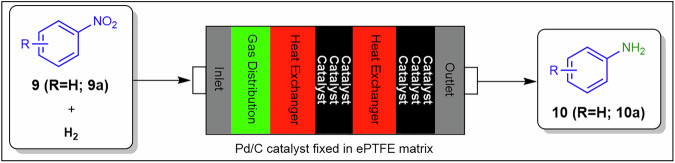
Continuous flow process for the preparation of **10**. Heterogeneous catalysis for the reduction (hydrogenation) of nitroarenes **9** to anilines **10** with the use of a novel modular packed-bed reactor^58^.

Breen et al.^58^ developed a novel, modular packed-bed reactor for heterogeneous catalysis, such as hydrogenation, to overcome the scalability and catalyst size limitations of traditional packed column reactors (Fig. 10). The reactor uses cartridges containing powdered catalysts embedded in an expanded ePTFE matrix. Its modular design allows catalytic layers and additional components to be configured for specific reaction conditions, desired results, and throughput requirements. Using this packed bed reactor, the authors demonstrated three classes of hydrogenation reactions: nitro reduction, debenzylation, and alkene reduction, achieving high or full conversion after optimisation. Embedding powders within an expanded ePTFE matrix mitigates wall adhesion and surface roughness effects, moderates pressure drop associated with bed compaction, and sustains scalable flow regimes despite catalyst particle heterogeneity

Reaction telescoping enables multiple reaction steps to be seamlessly linked in a single continuous process, improving efficiency and reducing processing time^73–75^. However, challenges arise when telescoping reactions due to reagent incompatibilities, where excess reagents or byproducts from one reaction step may interfere with or quench subsequent steps. To address these issues, in-line purification or scavenging systems are often incorporated^74,75^. These systems remove unwanted byproducts or residual reagents, ensuring compatibility between consecutive reaction steps and maintaining the overall efficiency and selectivity of the process.

**Fig. 11:**
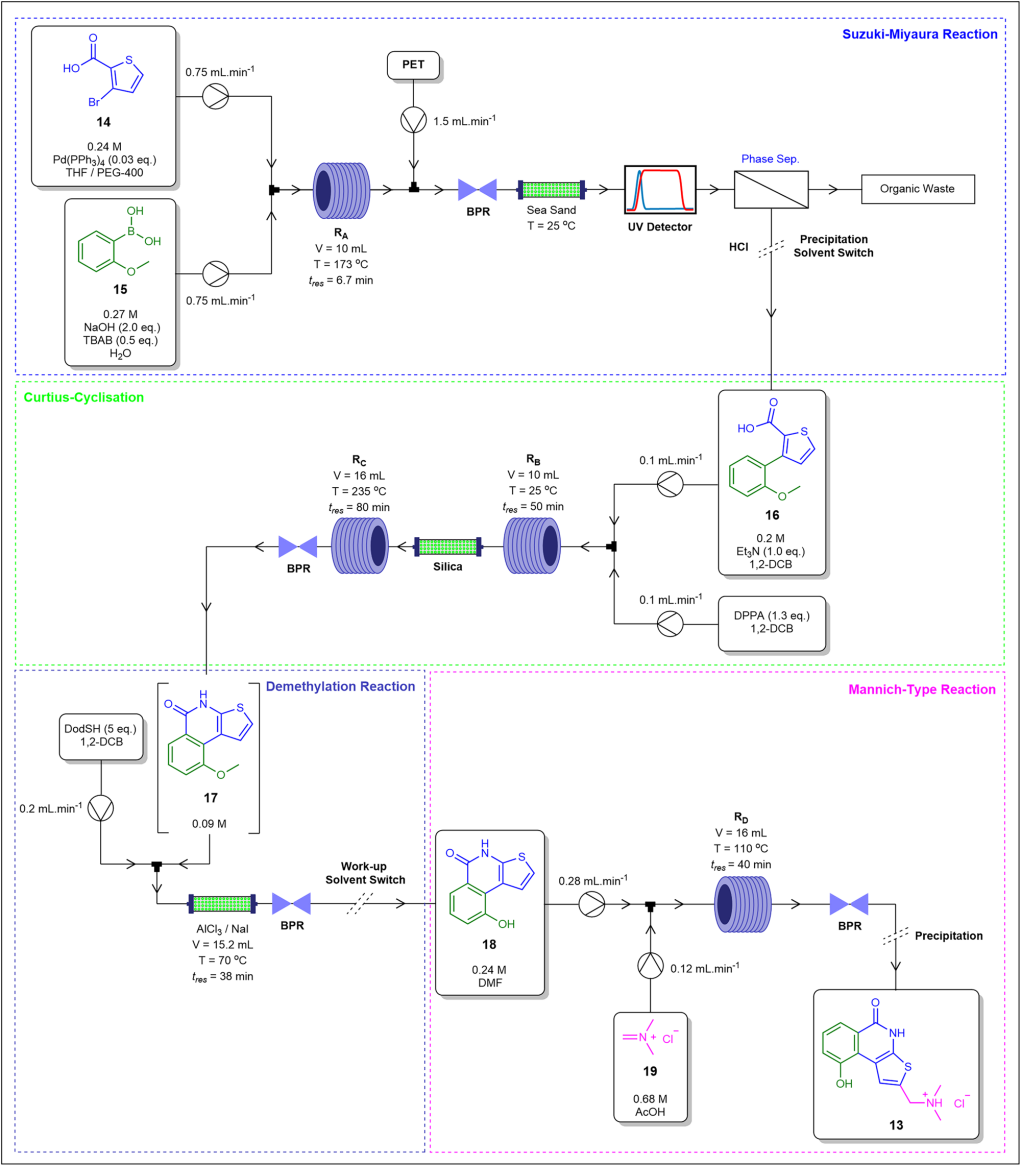
Telescoped flow process for the preparation of **13**. Telescoped continuous flow synthesis of 2-((dimethylamino)methyl)-9-hydroxythieno[2,3-c]isoquinolin-5(4*H*)-one hydrochloride **13** incorporating packed-bed reactors for both reaction execution and in-line purification^76^.

Filipponi and colleagues^76^ developed a four-step telescoped continuous flow synthesis, incorporating in-line purification and scavenging, to produce a PARP-1/2 inhibitor, compound **13** (Fig. 11), currently in preclinical trials for treating ischemia and inflammatory diseases. The process included a Suzuki-Miyaura reaction, a Curtius-cyclisation. a demethylation reaction, and a final Mannich-type reaction, with optimised conditions for each step detailed. The Suzuki-Miyaura reaction between thiophene derivative **14** and boronic acid **15** was conducted in a stainless-steel coil reactor, followed by a sea sand bed-packed reactor to remove palladium catalyst particles from the eluent flow. The resultant intermediate **16** was subjected to a subsequent Curtius-cyclisation with treatment of diphenylphosphoryl azide (DPPA) having occurred across three reactors, with the middle reactor containing a silica in a packed bed configuration to eliminate excess DPPA. In the demethylation step, intermediate **17** was treated with DodSH in an AlCl_3_/NaI packed-bed reactor to yield compound **18**. The final Mannich-type reaction took place in a stainless-steel coil reactor with the introduction of acetamide **19**, achieving an overall isolated yield of 55% with ≥97% purity. This work highlights the critical role of packed-bed reactor units in enabling both in-line purification and reaction facilitation, supporting efficient reaction telescoping under continuous flow conditions. Alternating packed-bed scavenging steps actively control precipitation of metal and reagent residues, stabilise pressure and residence-time profiles, and prevent fouling carryover between telescoped units.

Immobilised reagents, where active chemical species are anchored onto solid supports like resins, silica, or polymers, offer an effective strategy for handling poorly soluble solid reagents in continuous flow systems^77–79^. This approach minimises clogging, ensures controlled delivery of reactive species, and simplifies product separation, enhancing process efficiency and sustainability. Typically deployed in packed-bed reactors, monolithic columns, or fixed-bed configurations, immobilised reagents provide precise control over reaction conditions, improving mass and heat transfer compared to batch processes^77,78^. By preventing free solid movement, these systems reduce blockages while maintaining high reactivity, making them ideal for multiphase reactions in organic synthesis, catalysis, and pharmaceutical production. Additionally, immobilised reagents enable catalyst or reagent recycling, aligning with green chemistry principles by reducing waste and costs. It is important to note that using supported or unsupported solid reagents introduces engineering challenges as the contents of the packed-bed reactor change over time. When the reagent is gradually consumed and incorporated into the liquid phase, stoichiometry shifts occur, making steady-state operation difficult and potentially causing pressure drop.

**Fig. 12:**
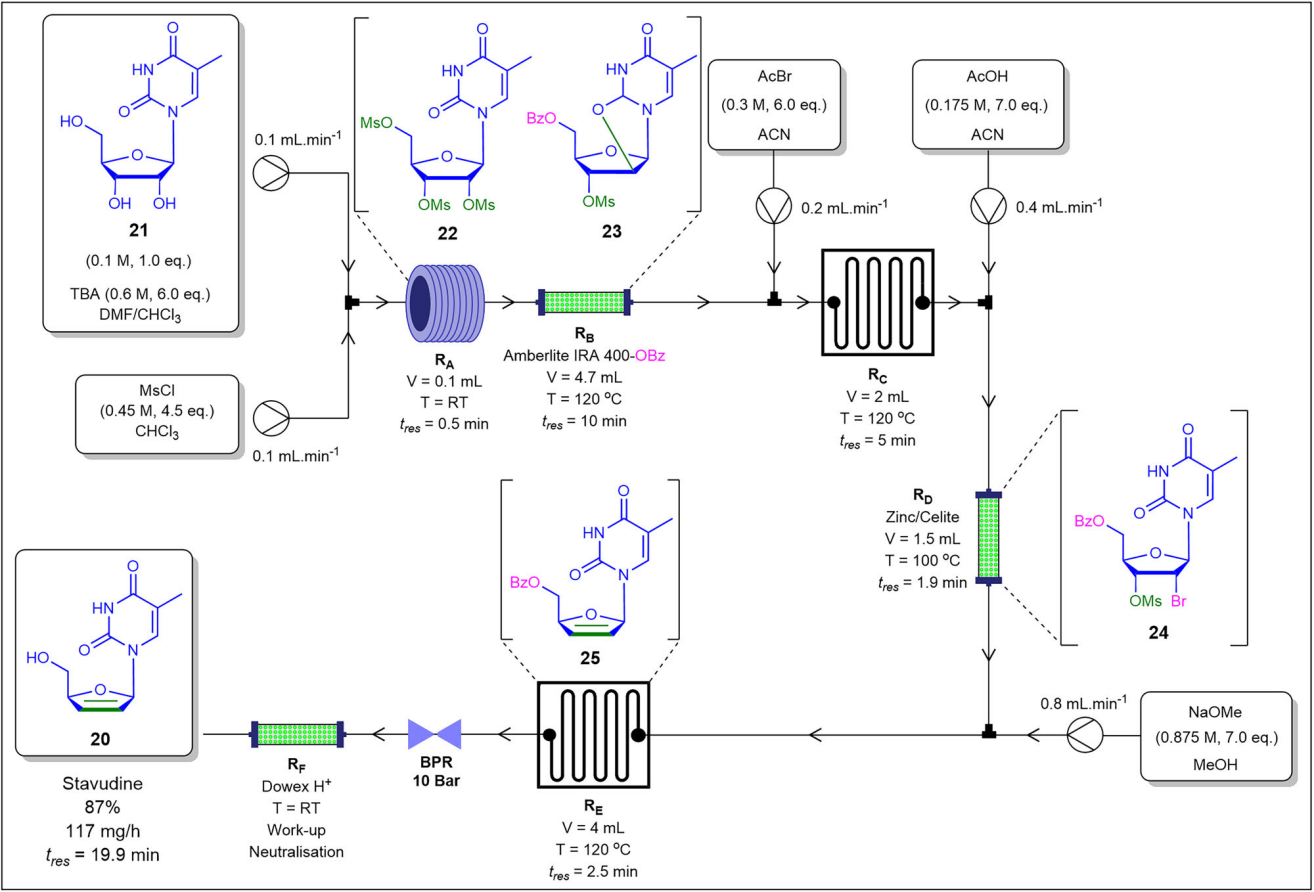
Continuous flow process for the preparation of Stavudine **20**. Continuous flow multistep synthesis of stavudine **20** involving packed bed reactors, mixing chips and coil reactors.

Sagandira and co-workers^74^ developed a six-step continuous flow synthesis of stavudine **20** from 5-methyluridine **21**, achieving an 87% overall yield with a total residence time of 19.9 min and a throughput of 117 mg.h^−1^ (Fig. 12). The process utilised three tubular reactors and three packed-bed reactors, performing trimesylation, cyclisation, 5′-benzoyl protection, 2′-bromination, 2′,3′-reductive elimination, and benzoyl deprotection in sequence with the synthetic intermediates **22** through **25** generated and consumed in situ. Due to the low solubility of sodium benzoate in organic solvents required for 5′-benzoyl protection and intermolecular cyclisation to synthesize 5′-benzoyl-3′-methanesulfonyl-2,2′-anhydro-5-methyluridine **23** from 2′,3′,5′-tris(methanesulfonyl)-5-methyluridine **22** under continuous flow conditions, the authors immobilised benzoate ions on Amberlite IRA 400 resin, enabling a clog-free and efficient continuous flow reaction. The 2′,3′-reductive elimination step was performed in a packed-bed reactor containing Zn/Celite and the Dowex H+ packed-bed reactor was used to neutralise the final eluent flow. Ion-exchange immobilisation converts a poorly soluble base into a fixed phase, eliminating salt precipitation within channels, reducing wall adhesion, and enabling liquid dosing via piston or syringe pumps without the instability associated with slurries.

Packed bed reactors remain a highly effective and widely adopted technology in continuous flow systems, as evidenced by numerous case studies demonstrating their versatility in handling solid reagents, catalysts, and in-line purification or scavenging systems. Scalability poses a significant challenge, as larger reactor sizes can worsen non-uniform flow patterns, like channelling, leading to reduced efficiency and inconsistent product quality. Engineering solutions, including the use of diffusers, can help mitigate these issues. High-pressure drops across the packed bed, especially in systems with fine catalyst particles or elevated flow rates, present additional processing difficulties. Ongoing research is essential to address these limitations and unlock the full potential of packed bed reactors in continuous flow systems.

### Flow mechanochemistry

Flow mechanochemistry is an emerging approach that integrates mechanical activation with continuous processing to enable efficient transformation of solids without reliance on bulk solvents. The technology is particularly effective for solid-state chemical synthesis, offering unique advantages in managing solids compared to traditional continuous flow systems^34,80,81^. It enables efficient handling and transformation of solids through mechanical forces and slurry streaming^82–84^. Unlike conventional slurry-based strategies, which often struggle with sedimentation, viscosity-driven pressure drops, and agglomeration, mechanochemical platforms such as twin-screw extruders (TSE) and jacketed single-screw reactors (JSSR) operate under controlled shear and temperature conditions to convert solid or paste feeds into reactive streams^34,80–84^. This capability makes mechanochemistry particularly valuable for solvent-lean or solvent-free synthesis, offering a sustainable route for reactions that are otherwise challenging in liquid-phase flow systems. While mechanochemistry differs in scope from traditional continuous flow methods designed for solids suspended in liquids, it serves as a complementary upstream technology that broadens the operational envelope of flow chemistry, enabling both to coexist within a unified and integrated manufacturing paradigm^80,82,84^. By enabling direct processing of solids and facilitating integration with telescoped sequences, mechanochemistry offers a strategic, scalable, and sustainable solution for complex synthetic routes in pharmaceutical and specialty chemical manufacturing. Mechanochemical platforms directly address rheological challenges such as paste viscosity, yield stress, and dynamic behaviours including agglomeration under shear, while avoiding precipitation in narrow channels by processing solids as the primary feed.

Although flow mechanochemistry is also employed in downstream processes such as crystallisation and precipitation, this discussion will focus solely on their application in upstream solid handling for reactions

**Fig. 13:**
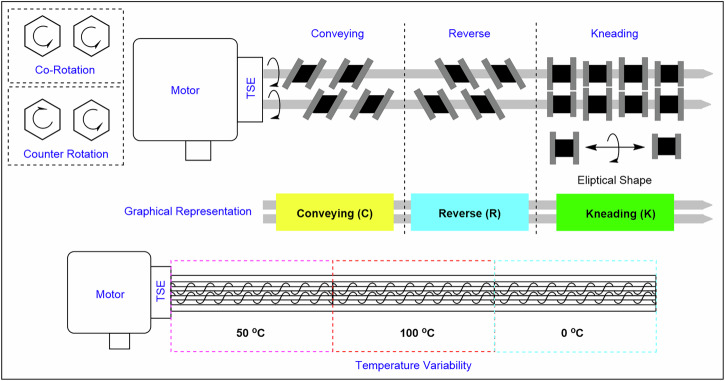
Illustration of a twin-screw extruder (TSE). Simplified illustration showcasing various screw configurations (conveying, kneading and reverse) and temperature variability across different sections of the reactor^34^.

One example of mechanochemistry technology is the twin-screw extruder (Fig. 13), which features a reaction centre configurable for conveying, reverse, or kneading operations through plug-and-play functionality^34^. Controlled kneading and conveying elements counter shear-induced agglomeration and maintain workable paste rheology, while integrated solid/liquid feeders eliminate the limitations of slurry pumping. The extruder is equipped with a motor that adjusts speed and torque, enabling fine-tuned mechanical control. Reagents are continuously introduced and removed at opposite ends of the extruder via continuous flow pumps^34,85,86^. Depending on the reactor design, operations can occur at varied temperatures, with some systems supporting multiple temperature zones, which is particularly useful for processes such as crystallisation^34,87^. For a comprehensive overview of advancements in this field, Browne and co-workers have reported a detailed review on continuous flow mechanochemistry^34^.

**Fig. 14:**
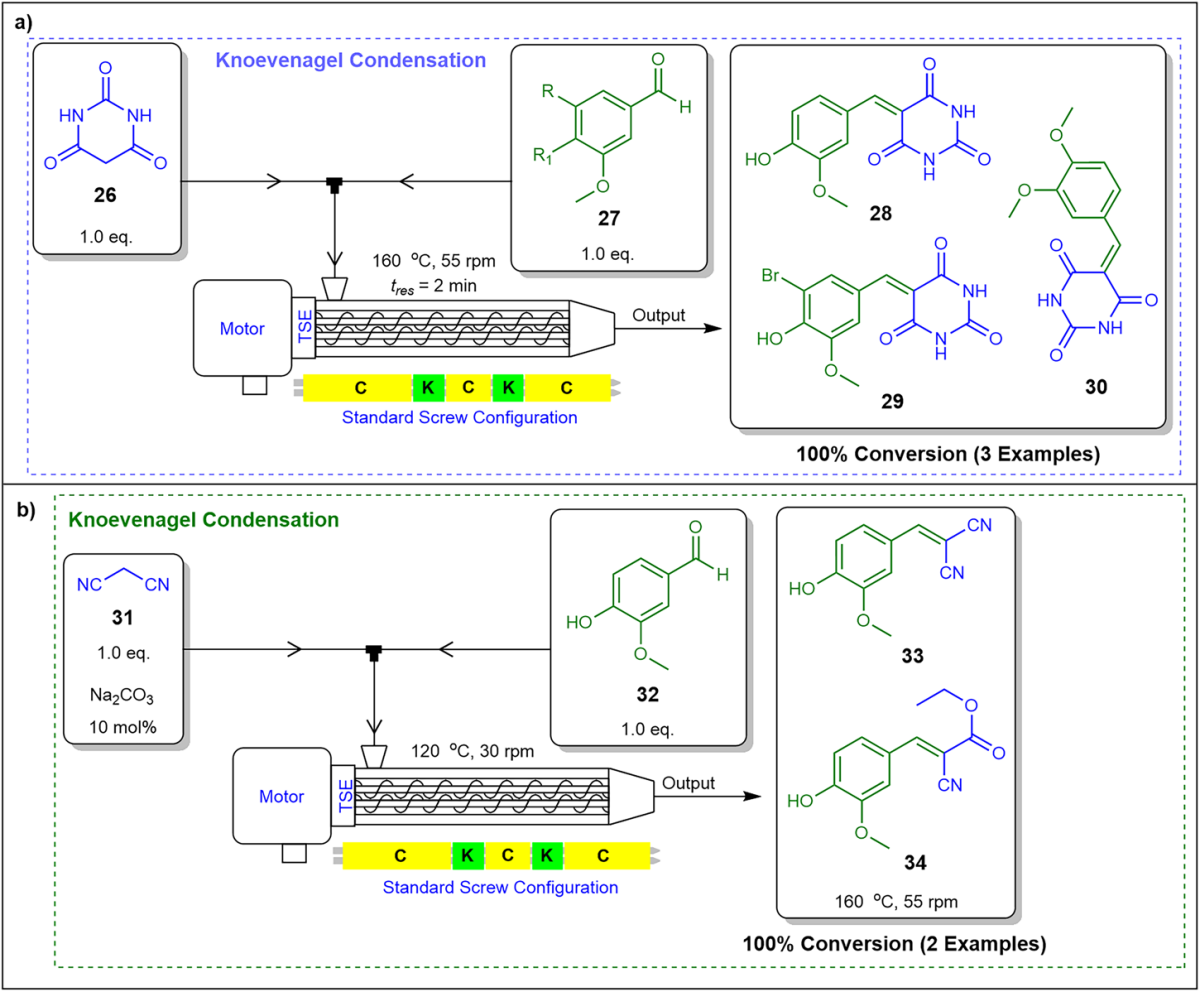
Implementing a twin-screw extruder for Knoevenagel condensations. **a** Synthesis of compounds **28,** **29** and **30** utilising a twin-screw extruder. **b** Synthesis of compounds **33** and **34** with the implantation of a twin-screw extruder^86^.

Extrusion equipment has been widely utilised across various chemistry disciplines beyond organic chemistry. The first organic chemistry application was reported in 2017 by Crawford and co-workers^86^, who adapted a Knoevenagel reaction involving barbituric acid **26** and benzaldehyde derivatives **27** from traditional ball-milling mechanochemistry to a continuous flow extrusion process (Fig. 14a). The reaction was conducted in a twin-screw held at 160 °C reactor with conveying and kneading screw elements rotating at 55 rpm and 2 min residence time. Under optimised conditions, the process achieved a throughput of 0.52 kg.h^−1^ and a space-time yield (STY) of 258,385 kg.m⁻³.day⁻¹, significantly surpassing the STY of conventional batch mechanochemistry at 40 kg.m⁻³.day⁻¹. Importantly, the preparation of **28,** **29**, and **30** were reported as having achieved 100% conversions^86^.

Building on their successful adaptation of a Knoevenagel reaction of **26** and **27** to continuous flow extrusion, Crawford and co-workers^86^ further explored the reaction of malononitrile **31** with vanillin **32** and a catalytic amount of sodium carbonate (10 mol%) at 120 °C to prevent polymerisation, achieving a quantitative yield of dinitrile adduct **33** (Fig. 14b). Subsequently, the authors optimised conditions for the synthesis of compound **34** at 160 °C in the twin-screw extruder. This configuration resulted in quantitative conversion, with a STY of 55,650 kg.m⁻³.day⁻¹ and a throughput of 0.11 kg.h⁻¹^86^. For this process, **32** and sodium carbonate were introduced via a solid feeder, while ethyl cyanoacetate was delivered through a liquid feeder using a syringe pump, ensuring precise reagent addition and efficient mixing within the extruder^86^.

**Fig. 15:**
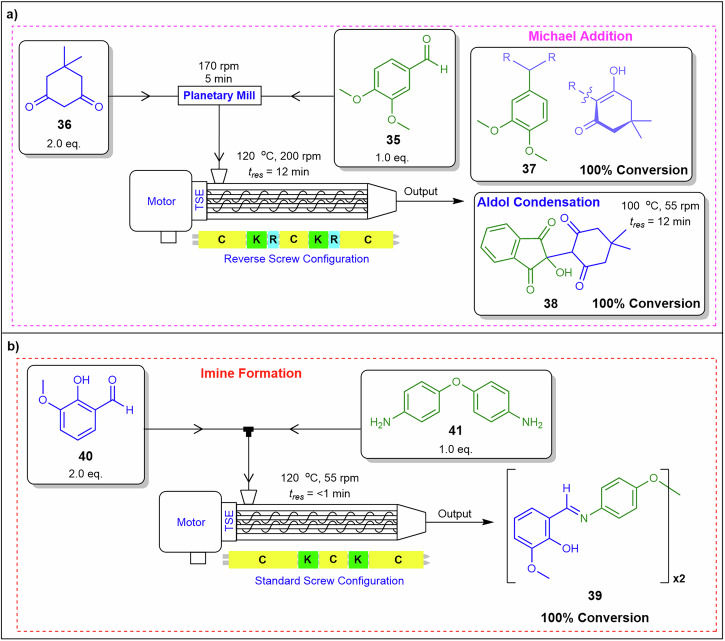
Implementing a twin-screw extruder for Michael addition, aldol condensation and imine formation. **a** Flow mechanochemistry system for Micheal addition and aldol condensation. **b** Flow mechanochemistry system for imine formation^86^.

Crawford and co-workers^86^ also demonstrated a Michael addition condensation reaction using 3,4-dimethoxybenzaldehyde **35** with dimedone **36** affording **37**, in addition to an aldol condensation with **36** and ninhydrin affording **38** (Fig. 15a). They systematically screened various parameters, including temperature, screw rotation speed, rotation style, and residence time, finding that lower temperatures and slower rotation speeds led to reduced conversions. The optimised conditions were determined to be 120 °C, a screw speed of 200 rpm, and 12 min residence time affording 100% conversion with a throughput of 0.070 kg.h⁻¹ and STY of 5000 kg.m⁻³.day⁻¹. The starting materials were pre-mixed using a planetary mill at 170 rpm for 5 min to ensure uniformity before being fed into the twin-screw extruder.

Crawford et al.^86^ utilised both ball-milling mechanochemistry and TSE to perform imine formation and afford di-imine **39** from starting materials **40** and **41**, employing a ball-milling frequency of 25 Hz and 30 min residence time (Fig. 15b)^86^. For the TSE process, optimised conditions included 120 °C, a screw rotation speed of 55 rpm, and a reagent feed rate of 0.79 g.min⁻¹. These conditions provided an STY of 14,900 kg.m⁻³.day⁻¹, significantly surpassing the performance of the corresponding batch process, which required extended reaction times^86,88^. Pre-mixing narrows particle size distributions, while screw speed and temperature setpoints adjust apparent viscosity to prevent channelling. Direct solid dosing further bypasses tubing diameter limitations typically associated with peristaltic delivery.

The success of the Knoevenagel condensation, Micheal addition, aldol condensation and amine formation in a twin-screw extruder has broader implications for organic synthesis, paving the way for other solid-state reactions to be adapted to continuous flow systems. It also addresses common challenges in solid handling, such as blockages or uneven mixing, by optimising pre-mixing and screw configurations. This work by Crawford et al.^86^ serves as a benchmark for the integration of extrusion technology in organic chemistry, offering a robust, efficient, and sustainable alternative to conventional mechanochemical methods.

**Fig. 16:**
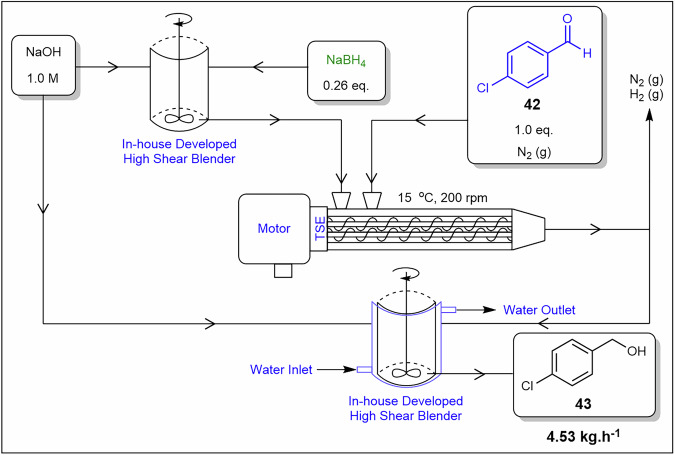
Continuous flow process for the preparation of **43** using twin-screw extrusion. Continuous flow schematic displaying the mechanochemical reduction of 4-chlorobenzaldehyde **42**^89^.

To demonstrate scalability of flow mechanochemistry, Isoni and co-workers^89^ developed a continuous, multi-kilogram-scale system for reducing solid aldehydes using a shear reactor (Fig. 16). On a gram scale, initial experiments involved reducing aldehydes under solvent-free conditions at a high shear frequency of 550 W and a rotation speed of 14,000 rpm, achieving conversions ranging from 25–95% in less than 1 min with conversion rates enhanced through the addition of NaOH (1.0 M)^89^. At kilogram-scale, 4-chlorobenzaldehyde **42** was successfully reduced in a modified twin-screw extruder using a NaOH/NaBH₄ (1.0 M) slurry under a nitrogen atmosphere, yielding 89% of product **43** in a 17 min run time and throughput of 4.53 kg.h⁻¹. Despite these impressive results, the process had two challenges: fouling of the NaBH₄ slurry nozzle and an uncontrolled temperature increase of 15 °C. However, the system offered significant advantages, including the elimination of organic solvents, improved volume efficiency, enhanced energy efficiency, and reduced operational costs. The observed nozzle fouling is attributed to precipitation and adhesion, while high shear mitigates sedimentation. Solvent-free operation eliminates viscosity drawbacks associated with slurries but requires thermal management to maintain stable rheology.

**Fig. 17:**
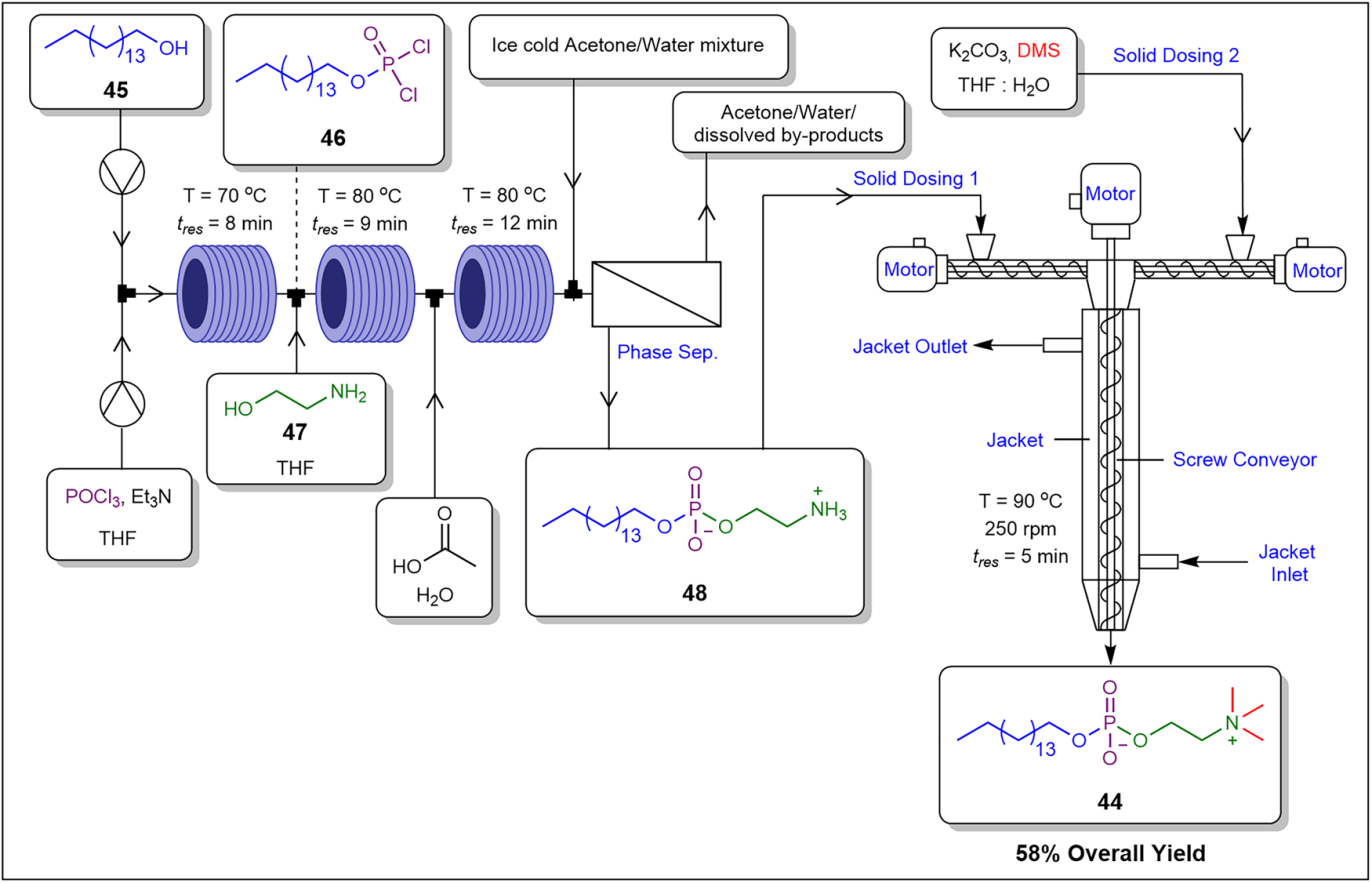
A twin-screw extrusion enabled preparation of Miltefosine **44**. Telescoped 4-step continuous flow preparation of Miltefosine **44** involving a mechanochemical step in a screw reactor^90^.

Patil et al.^90^ and Sharma et al.^30^ developed several microfluidic mechanochemical platforms utilising a jacketed single-screw reactor (JSSR) designed to handle solids and viscous slurries. The authors demonstrated a telescoped 4-step continuous flow synthesis of miltefosine **44** involving a mechanochemistry step (Fig. 17)^90^. In the first step, hexadecanol **45** was chlorinated with POCl₃ in a coil reactor at 70 °C with an 8-min residence time, yielding hexadecyl phosphorodichloridate **46** in 85% yield in situ. The eluent flow was treated with ethanolamine **47** in the second coil reactor, followed by a reaction with acetic acid in the third coil reactor to produce 2-ammonioethyl hexadecyl phosphate **48** (Fig. 17). The resulting product was washed with acetone/water mixtures, forming a solid paste. The phosphate **48** paste was fed into the JSSR, where it reacted with dimethyl sulfate in the presence of potassium carbonate at 90 °C, with the screw rotating at 250 rpm for 300 s, to yield miltefosine **44** in 58% overall yield and 10 g.h^−1^ throughput. The total residence time was 34 min, a significant improvement over the 15-h duration required for the equivalent batch process^86,90^. Feeding a solid paste directly engages the rheological limits of the screw reactor and applies controlled shear to prevent agglomeration and settling, creating a solids-tolerant interface between liquid-phase units and upstream mechanochemical conversion.

All the reviewed case studies highlight the practical advantages of flow mechanochemistry for handling solids under continuous flow conditions. By leveraging mechanical forces and continuous processing, the flow mechanochemistry reactors facilitate efficient solid mixing, eliminate the need for excessive solvents, and minimise manual handling, aligning with green chemistry principles. The high throughput and excellent STY underscore its potential for industrial scalability, particularly for solid-state reactions that are challenging in traditional solution-based or batch systems.

### Pickering emulsion and colloidal nano-particle suspensions

**Fig. 18:**
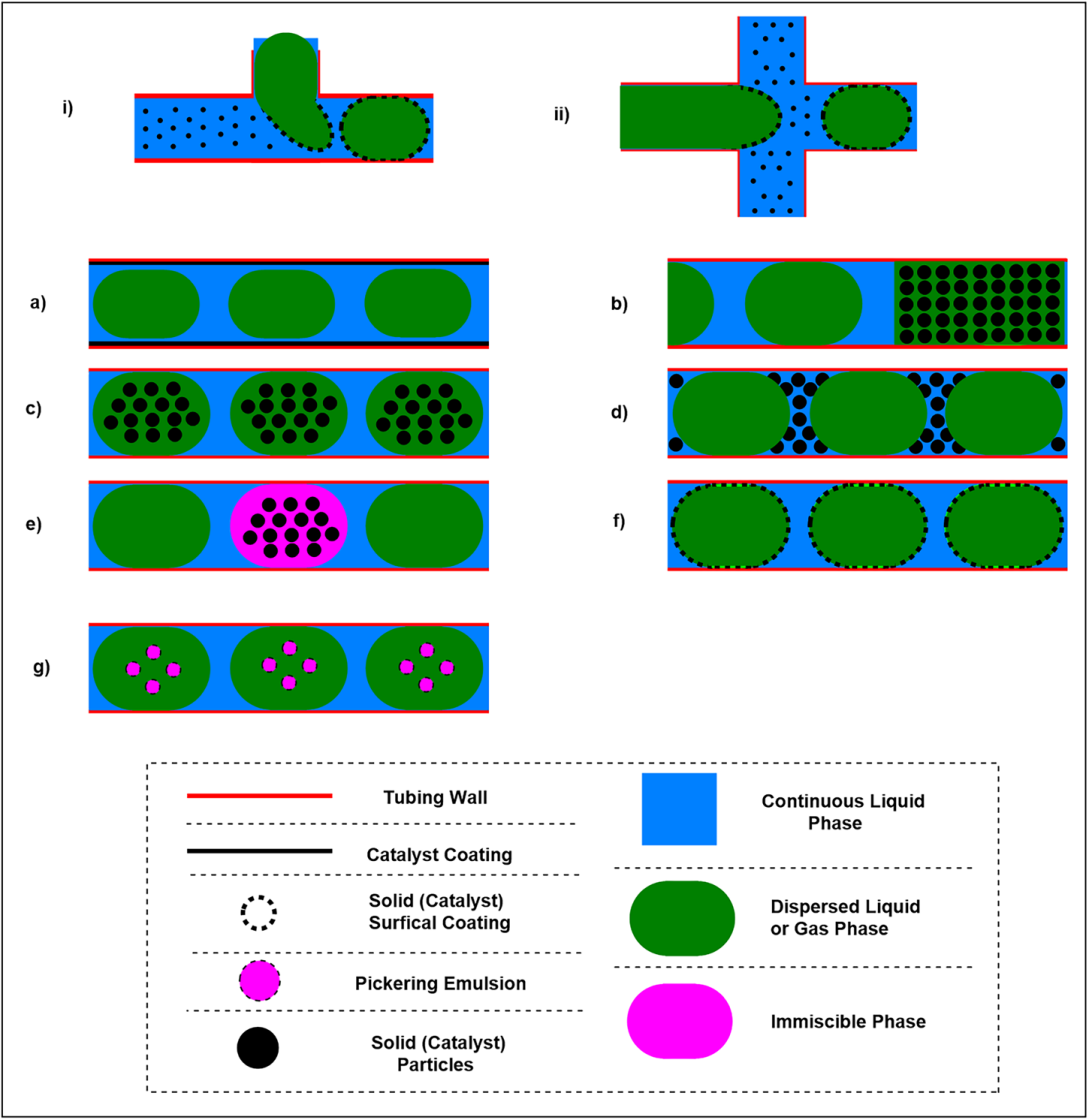
Multiphase reactions performed in continuous flow modes. **a** Catalyst-coated inner wall of tubing; **b** Packed-bed reactor; **c** Solid particle (catalyst) dispersion in an immiscible phase; **d** Solid particle (catalyst) dispersion in continuous liquid phase; **e** Solid particle (catalyst) dispersion in another immiscible phase separated by gas bubbles; **f** Pickering emulsion droplets with solid (catalyst) particles hosted within the continuous phase; **g** Pickering emulsion droplets with solid (catalyst) particles hosted in another immiscible phase, all within the continuous phase.

Another effective strategy for handling solids in continuous flow systems involves the use of Pickering emulsions (PEs), which are emulsions stabilised by solid particles (100 μm to 10 nm) adsorbed at the interface of two immiscible liquids, forming PE droplets with sizes between 10 nm and 1000 μm (Fig. 18)^31,91^. This technique is widely applied across various industries, particularly in heterogeneous catalysis, due to its high efficiency^31,92,93^. In continuous flow systems, PEs are prepared using T-piece, cross-mixing, or flow-focusing input mixing units. In these setups, a slurry containing solid suspensions is mixed with a continuous fluid stream, creating a segmented regime characterised by alternating segments of solid-suspended material and the carrier fluid (Fig. 18i, ii)^31^. Compared to other solid-handling techniques in flow chemistry, PEs offer several advantages: they require no additional external energy, provide precise control over droplet size, minimise or eliminate unwanted pressure drops, and do not rely on complex mechanical equipment^31,94^. PEs can be synthesised and mobilised prior to the reaction or directly within a microfluidic platform, resulting in a biphasic system with either a liquid–liquid slug flow or a monodispersed droplet regime (Fig. 18f)^31,93,95^. There are diverse options available to modern flow chemists for conducting multi-phase reactions, ranging from catalyst wall coatings (Fig. 18a) to Pickering emulsions (Fig. 18g)^31^. Interfacial stabilisation reframes precipitation and fouling risks by localising solids at liquid–liquid boundaries, while nanoscale mobility minimises sedimentation and blockage in narrow channels. This approach remains compatible with peristaltic delivery provided particle size meets tubing constraints.

Colloidal suspensions of nanoparticles, closely related to Pickering emulsions (PEs), have garnered significant interest in continuous flow chemistry due to their exceptional mobility within a continuous flow stream, attributed to their nanoscale particle sizes (typically 1–100 nm). These suspensions offer unique advantages for catalytic reactions, combining the high catalytic activity typical of homogeneous catalysis with the practical benefits of heterogeneous catalysis, such as simplified solid catalyst separation and recovery during work-up procedures^31,96,97^. Both PEs and colloidal nanoparticle suspensions excel in their compatibility with a wide range of continuous flow chemistry techniques. They can be seamlessly integrated into applications such as heterogeneous photocatalytic reactions, porous packed-bed reactors, pollutant degradation, heterogeneous reductions, water electrolysis, heterogeneous catalysis, and visible-light photo-redox reactions, among others^31,96–125^. The adaptability of colloidal suspensions and PEs, combined with their capacity to improve reaction efficiency and facilitate catalyst recovery, makes them highly effective tools for promoting sustainable and scalable chemical processes, particularly in managing solids within continuous flow systems.

**Fig. 19:**
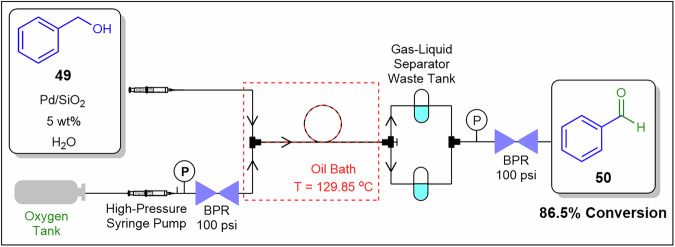
Continuous flow process for the preparation of **50**. Continuous flow oxidation of **49** to benzaldehyde **50** using the Pickering emulsion technique^126^.

Li et al.^126^ reported a PE system in continuous flow for the oxidation of benzyl alcohol **49** to benzaldehyde **50**, demonstrating effective solid handling in a triphasic heterogeneous system (Fig. 19). Pd/SiO_2_ nanoparticles served as the catalyst, introduced with the continuous phase of either benzyl alcohol or water and mixed with the dispersed phase of either water or benzyl alcohol to form either an oil-in-water or water-in-oil PE. The mixture was combined with O_2_ (g) via a T-piece unit and subsequently flowed through a tube reactor for the oxidation. Optimised conditions included 129.8 °C, a 5 wt% catalyst-to-benzyl alcohol ratio, and an O_2_-to-benzyl alcohol molar ratio of 0.76, yielding 86.5% conversion and 99.9% selectivity for benzaldehyde **50**. Solid nanoparticles anchored at droplet interfaces suppress wall adhesion and aggregation, while controlled droplet sizing optimises mass transfer and pressure drop without relying on high-viscosity slurries.

**Fig. 20:**
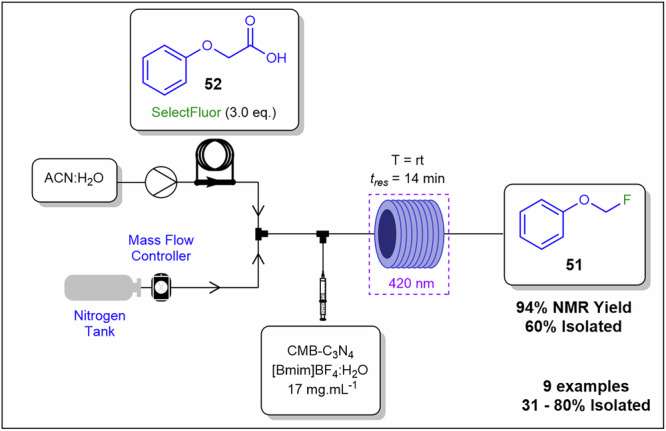
Continuous flow process for the preparation of **51**. Continuous flow schematic for the preparation of **51** with the use of CMB-C_3_N_4_ in serial micro-batch reactors (SMBR).

Pieber and co-workers^98^ developed an innovative approach to handle solids in continuous flow by employing a solid catalytic suspension in a gas–liquid flow system, resembling a series of segmented micro batch reactors (SMBRs). The system was established by mixing an inert gas (nitrogen, regulated by a mass flow controller) with a reaction solvent using a Y-piece mixing unit, followed by the introduction of a solid catalytic suspension via a T-piece mixer equipped with a syringe pump and a magnetic stirrer bar for uniform dosing. The triphasic reaction mixture was processed in a coil reactor, housed in either a photochemical reactor or a thermostatic bath (Fig. 20)^98^. This system was evaluated for decarboxylative fluorination of phenoxy acetic acids and fluorination of various compounds. Reactants, including carboxylic acid-bearing compounds, were delivered in an acetonitrile-water solution via a sample loop, while the catalyst, carbon nitride (CMB-C_3_N_4_), was suspended in a solution of 1-butyl-3-methylimidazolium tetrafluoroborate ([Bmim]BF_4_) and water. The magnetic stirrer ensured consistent catalyst dispersion, and internal vortices within the SMBRs promoted system uniformity^98,127^. Substrate scope studies demonstrated a range of yields with fluoride containing **51** as a represented example of 60% isolated yield from phenoxyacetic acid **52** as the synthetic precursor, demonstrating the system’s versatility. Scalability tests yielded positive results, and recyclability studies highlighted the high reusability of CMB-C_3_N_4_ and the ability to recycle [Bmim]BF_4_. In contrast, prior studies using [Ru(bpy)_3_]^2+^ as a catalyst reported inferior performance and system clogging, underscoring the advantages of the CMB-C_3_N_4_-based approach^98^. Gas–liquid segmentation enforces uniform residence times despite non-Newtonian behaviour, prevents sedimentation and bridging, and limits particle–wall contact to reduce fouling, while remaining compatible with peristaltic feed constraints.

**Fig. 21:**
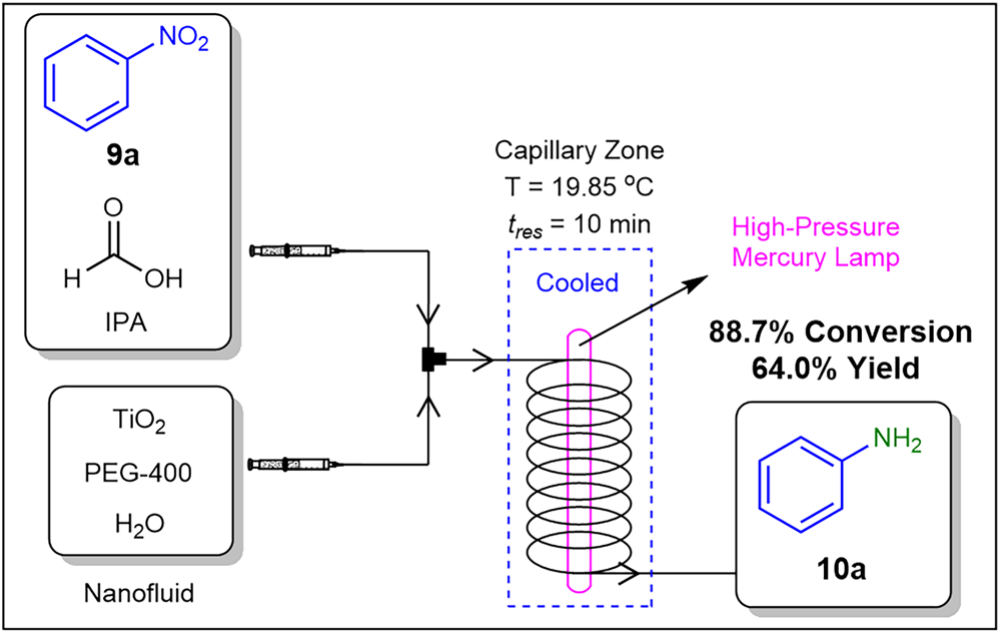
Continuous flow process for the preparation of **10a**. A continuous flow photocatalytic nitro-reduction of aniline made possible with the utilisation of a TiO_2_ nanofluid^128^.

Pu and co-workers^128^ demonstrated a TiO_2_ nanocatalyst photocatalytic approach for nitrobenzene reduction to aniline under continuous flow conditions (Fig. 21). The TiO_2_ nanocatalyst was used as a nanofluid prepared in PEG-400 and deionized water. As a model reaction, they reduced nitrobenzene **9a** to aniline **10a** under UV irradiation from a high-pressure mercury lamp. The optimised conditions included a catalyst-to-nitrobenzene mass ratio of 0.4 (catalyst concentration of 6.6 mg.mL⁻¹) and a 10-min residence time, achieving an 88.7% conversion and a 64.0% yield of aniline **10a**. Catalyst reusability studies demonstrated excellent photocatalyst stability over four consecutive reaction cycles. Additionally, the photo-microreactor exhibited a high photonic efficiency during this continuous heterogeneous catalytic process. Nanoscale particles stabilise suspensions by reducing settling, while solvent selection controls viscosity to limit pressure drop and wall adhesion in microchannels.

**Fig. 22:**
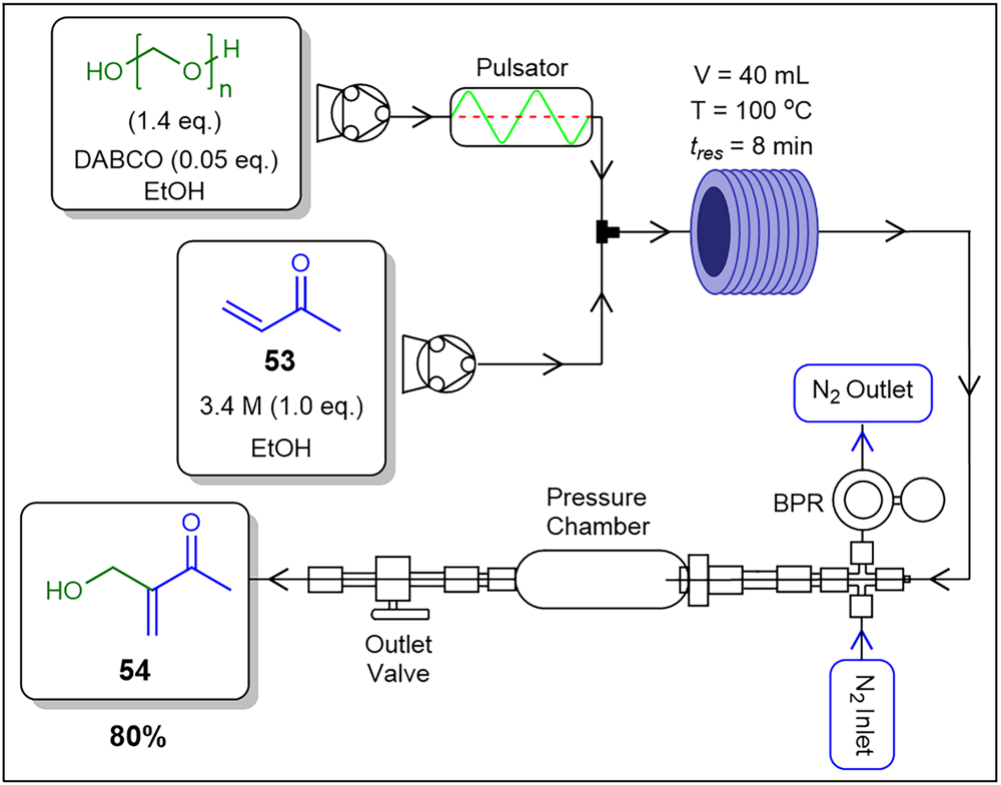
Flow process for the preparation of **54**. Continuous flow α-hydroxymethylation of methyl vinyl ketone **53** using formaldehyde gas generated in situ from the paraformaldehyde slurry^129^.

*α*-Hydroxymethylation reactions are vital in the pharmaceutical industry, with formaldehyde as a key reagent. However, aqueous formaldehyde limits compatibility to water-tolerant substrates, while gaseous formaldehyde is avoided due to its toxicity, hazards, and need for excess quantities. Paraformaldehyde offers a safer, easier-to-handle alternative. Vandekerckhove and co-workers^129^ developed a continuous flow method for the α-hydroxymethylation of methyl vinyl ketone **53** using a paraformaldehyde slurry affording **54** (Fig. 22). In the presence of 1,4-diazabicyclo[2.2.2]octane (DABCO), paraformaldehyde is depolymerised in situ, generating and consuming formaldehyde gas within the system, enhancing safety and efficiency. This system effectively handled the paraformaldehyde slurry, outperforming other flow techniques like semi-continuous syringe pumps with internal mixing, which suffered from low formaldehyde output and solid sedimentation^129^. The setup utilised a peristaltic pump connected to a diaphragm metering pump, modified into a pulsating unit (50% amplitude) by removing its valves and equipped with a frequency controller to achieve oscillatory flow at 3 Hz. The eluent stream passed through a blockage-resistant back-pressure regulator. Nitrogen gas pressurised the vessel via Swagelok connections, supporting up to 100-bar pressure. Optimised conditions yielded product **54** in 80% isolated yield and 81.6 g.h⁻¹ throughput, significantly surpassing the batch productivity of 0.06 g.h⁻¹. Pump design and oscillatory flow strategies mitigate slurry rheology challenges and pulsation-induced instabilities, while particle-to-tubing size ratios reduce blockage risk and in situ depolymerization governs precipitation pathways.

**Fig. 23:**
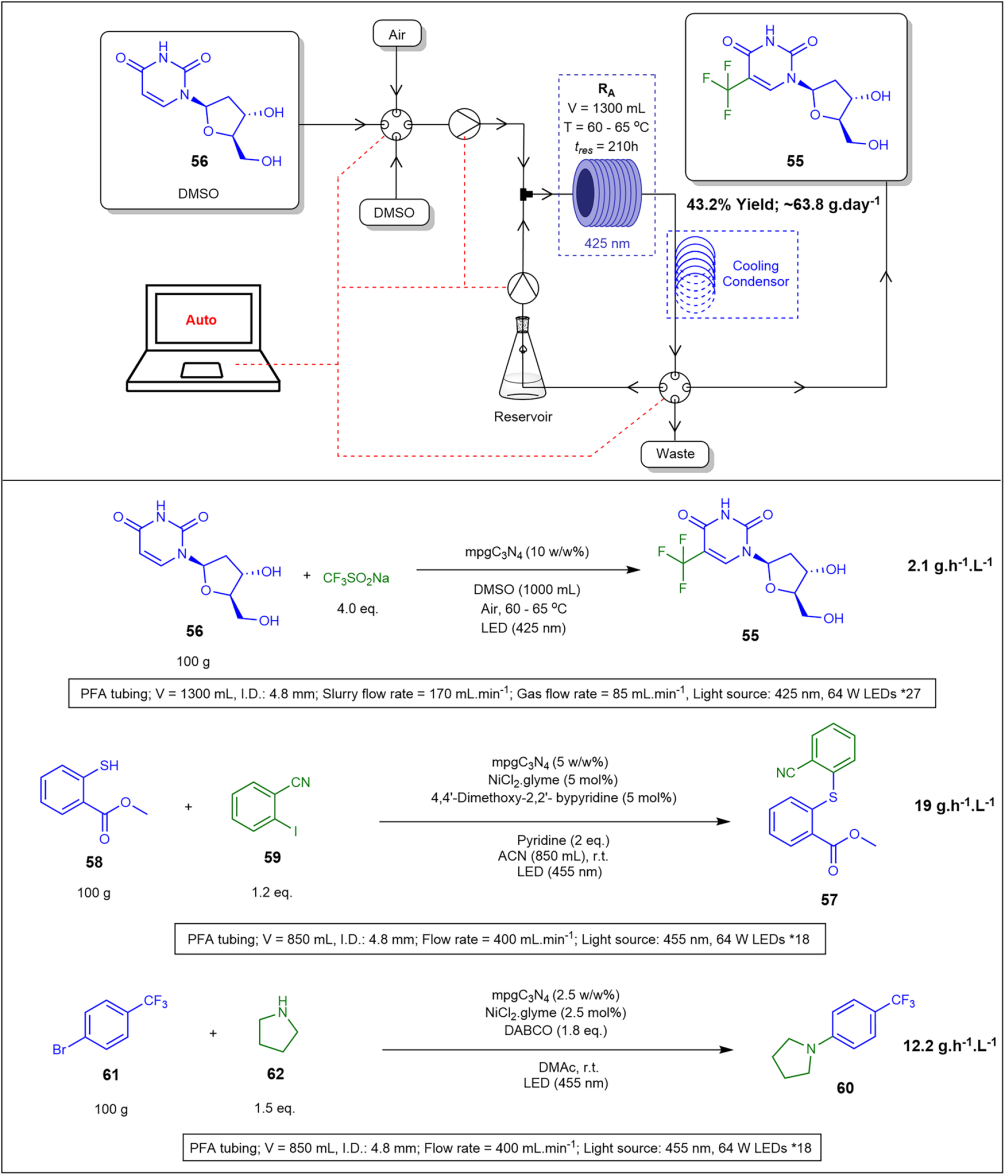
Continuous flow heterogeneous photoreactions. Reaction schematic highlighting the multiple products obtained while using a high-speed circulation flow mode for C–C, C–N and C–S cross-coupling reactions to synthesis **55,** **57** and **60**^130^.

Continuous flow heterogeneous photoreactions face challenges such as solid handling, poor light penetration, and prolonged reaction times. Liu and co-workers^130^ addressed these issues by developing a high-speed circulation flow mode, which enhances mixing efficiency and prevents solid sedimentation, thus avoiding clogging. Using this approach, they conducted C–C, C–N and C–S cross-coupling reactions with a heterogeneous photocatalyst and nickel catalyst to synthesise trifluridine **55** from **56**, methyl 2-((2-cyanophenyl)thio)benzoate **57** from **58** and **59**, and 1-(4-(trifluoromethyl)phenyl)pyrrolidine **60** from **61** and **62** (Fig. 23). Compounds **57** and **60** were synthesised at 100 g-scale with productivity rates of 12.2 g.L⁻¹.h⁻¹ and 19 g.h⁻¹, respectively, and kilogram-scale synthesis of trifluridine **55** at 63.8 g.day⁻¹. A peristaltic pump was used to introduce air and a solid suspension slurry into the reactor system. This system outperformed conventional batch reactors and standard continuous flow systems, overcoming limitations such as clogging, reactor fouling, extended residence times, and reduced yields. This technology facilitates the transition from batch to continuous flow processes, overcoming solid-handling challenges and opening new avenues for organic synthesis^31,130^. High-velocity recirculation minimises sedimentation and agglomeration, enhances photon penetration in particle-laden streams, and stabilizes pressure and residence-time distribution in non-Newtonian flows, with peristaltic delivery optimised for particle size constraints.

From a production perspective, scaling these approaches requires precise control over droplet size and distribution, as variations can impact mass transfer and reaction kinetics. Catalyst recovery and reuse remain critical for economic and environmental viability, often achieved through phase separation or filtration strategies. Materials compatibility must be considered to prevent interfacial destabilisation under process conditions, while regulatory compliance for API campaigns demands robust validation of particle stability, reproducibility, and impurity control. Although laboratory-scale demonstrations are promising, translating these systems to industrial scale hinges on continuous monitoring of emulsion stability, integration with high-throughput mixing technologies, and standardized protocols for catalyst recycling and cleaning-in-place operations.

### Dynamic mixing reactor systems

Both academia and industry are actively advancing the design of reactors equipped with inherent solid-handling capabilities to address one of the major limitations of continuous flow processing. A central strategy involves the integration of dynamic mixing technologies directly into reactor architectures to minimise sedimentation, improve suspension stability, and prevent blockages. Examples include mechanically agitated systems such as continuous stirred tank reactors (CSTRs), sonication-assisted reactors that employ acoustic energy to maintain solids in suspension and spinning disc reactors that generate high shear forces to enhance mixing and mass transfer. These approaches not only improve reactor reliability and operational continuity but also expand the scope of reactions that can be performed under continuous flow conditions, thereby facilitating the processing of traditionally challenging heterogeneous systems.

#### Continuous stirred tank reactors (CSTRs)

Continuous stirred tank reactors (CSTRs) have emerged as a robust solution, particularly for handling solids in continuous flow processes. CSTRs are highly effective in managing solids, such as precipitates or insoluble bases formed during reactions, and in facilitating the mixing of heterogeneous mixtures, making them a versatile tool across various flow chemistry applications^131,132^. CSTRs are distinguished by their exceptional mechanical agitation, which enables efficient handling of solid-forming reactions and accommodates long residence times with a broad residence time distribution (RTD)^132,133^. This agitation ensures uniform mixing and prevents sedimentation, making CSTRs ideal for reactions involving slurries or insoluble components. By connecting small CSTRs in series, process performance is enhanced, RTD is reduced, and active mixing is promoted through short inter-tank connections, making cascade CSTRs particularly suitable for continuous flow slurry handling^131^. This subsection reviews recent literature on the application of CSTRs for reaction solid handling in continuous flow transformations. While CSTRs are also employed in downstream processes such as crystallisation, this discussion will focus solely on their application in upstream solid handling for reactions. CSTR cascades directly target slurry rheology and broad RTDs, use agitation to suppress sedimentation and bridging, and reduce wall adhesion by preventing stagnant boundary layers.

**Fig. 24:**
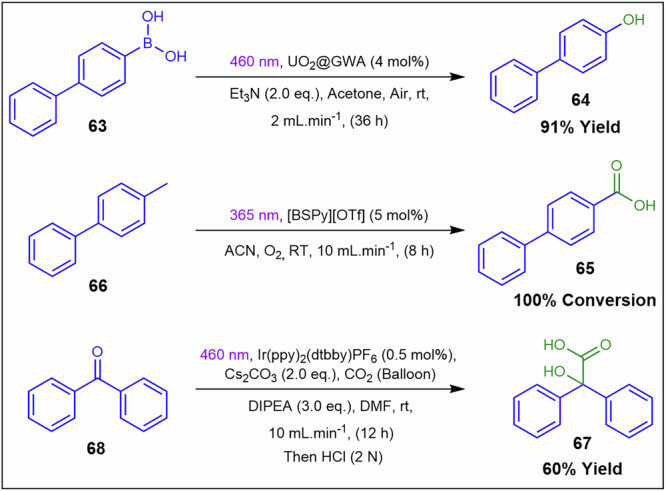
Reaction schematic for the preparation of **64**, **65** and **67** while using a heterogeneous photocatalytic approach. Heterogeneous photocatalytic preparation of 4-phenylphenol **64**, 4-phenylbenzoic acid **65** and carboxylic acid **67** in a CSTR^134^.

Ali and co-workers^134^ developed an 8-cell continuous stirred tank reactor (CSTR) optimised for heterogeneous photocatalytic reactions, featuring borosilicate glass cells with maximal photon flux, fitted with magnetic stirrer bars, heat probes, and LEDs (Fig. 24). The first reaction utilised uranyl-supported glass-wool (UO2@GWA) as a catalyst with blue LEDs (460 nm) to oxidise 4-phenylbenzeneboronic acid **63** to 4-phenylphenol **64**. Conducted at a 2 mL.min^−1^ flow rate (recirculated using a peristaltic pump) without stirring, the gram-scale reaction achieved a 91% yield over 36 h. Compared to batch processes, the system demonstrated superior performance, with catalyst recyclability up to 12 cycles, no clogging over 60 h, and high stability and reproducibility. Furthermore, the authors reported additional heterogeneous reactions for the preparation of 4-phenylbenzoic acid **65** from **66** with 100% conversion and carboxylic acid **67** from the diphenylketone precursor **68** with 60% yield (Fig. 24). These additional examples demonstrated as confirmation for the CSTR’s capability to handle biphasic and triphasic systems in both horizontal and vertical flow configurations. Magnetic stirring and recirculation minimise settling in triphasic streams, stabilise apparent viscosity, and limit fouling on transparent walls crucial for photon flux, with peristaltic recirculation matched to particle size and tubing constraints.

**Fig. 25:**
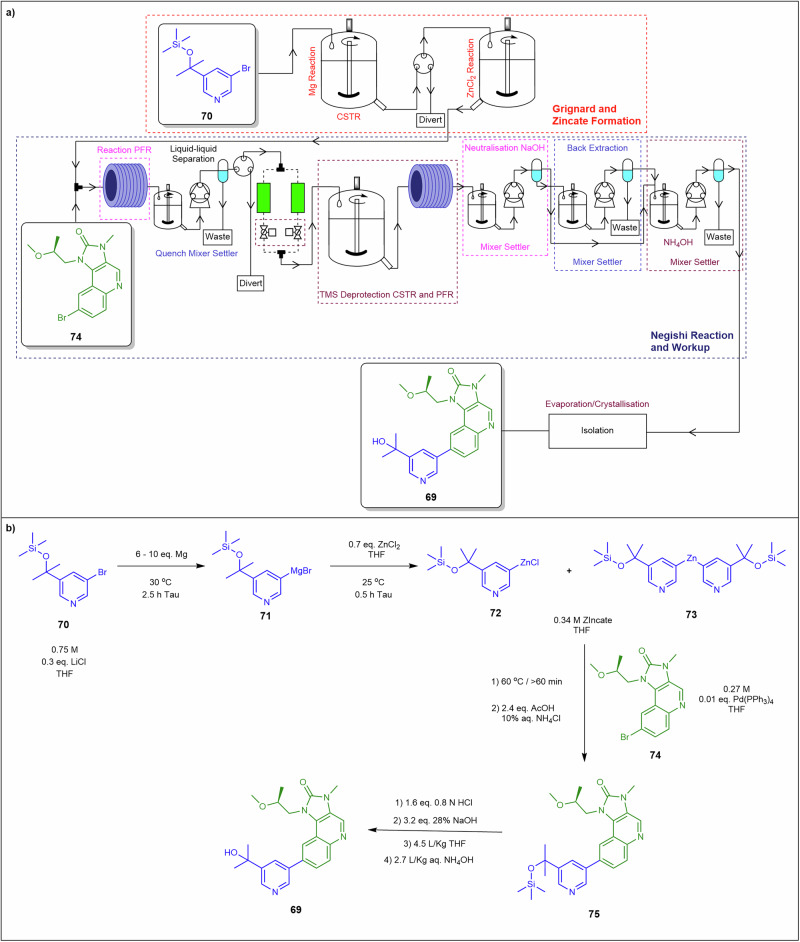
Flow process for the synthesis of API **69**. **a** Continuous flow system for Grignard reagent/Zincate synthesis and Negishi coupling reactions to prepare API **69**. **b** Synthetic route to API 69 from **70** ^135^.

Calvin et al.^135^ developed a continuous flow process for synthesising active pharmaceutical ingredient (API) **69** through a telescoped multi-step reaction sequence (Fig. 25). The process began with a Grignard formation, where TMS-protected compound **70** and LiCl was introduced into a CSTR containing magnesium at 30 °C, with a 2.5-h residence time. This setup ensured effective control of the heterogeneous reaction, minimising degradation impurities and yielding Grignard reagent **71**. This stream was then fed into a second CSTR, where it reacted with ZnCl₂ at 25 °C for 0.5 h, forming zincate intermediates **72** and **73**. These intermediates were directed to a plug flow reactor at 60 °C for 1 h, undergoing a Negishi coupling with quinoline bromide derivative **74** and Pd(PPh₃)₄, affording full conversion to TMS-protected API **75**. The final deprotection step involved treating the organic stream containing **75** with HCl in a CSTR for 30 min, followed by an additional 25 min in a plug flow reactor, resulting in complete deprotection. The stream was then neutralised with NaOH in a CSTR, followed by an NH₄OH wash in another CSTR to remove zinc byproducts, water, and most palladium. The aqueous layer underwent back-extraction with THF in a separate CSTR, and the combined organic layers were washed again with NH₄OH in a CSTR. The crude organic stream was collected in surge drums for distillation and crystallisation, yielding ~13 kg.day⁻¹ of crude API **69** in solution over 3–4 weeks of continuous operation. Post-crystallisation, over 200 kg of purified API **69** was isolated with >99.95% purity. CSTRs tolerate insoluble metal residues and salt formation, control slurry viscosity and pressure drop across telescoped units, and limit adhesion via active mixing that disrupts boundary fouling.

**Fig. 26:**
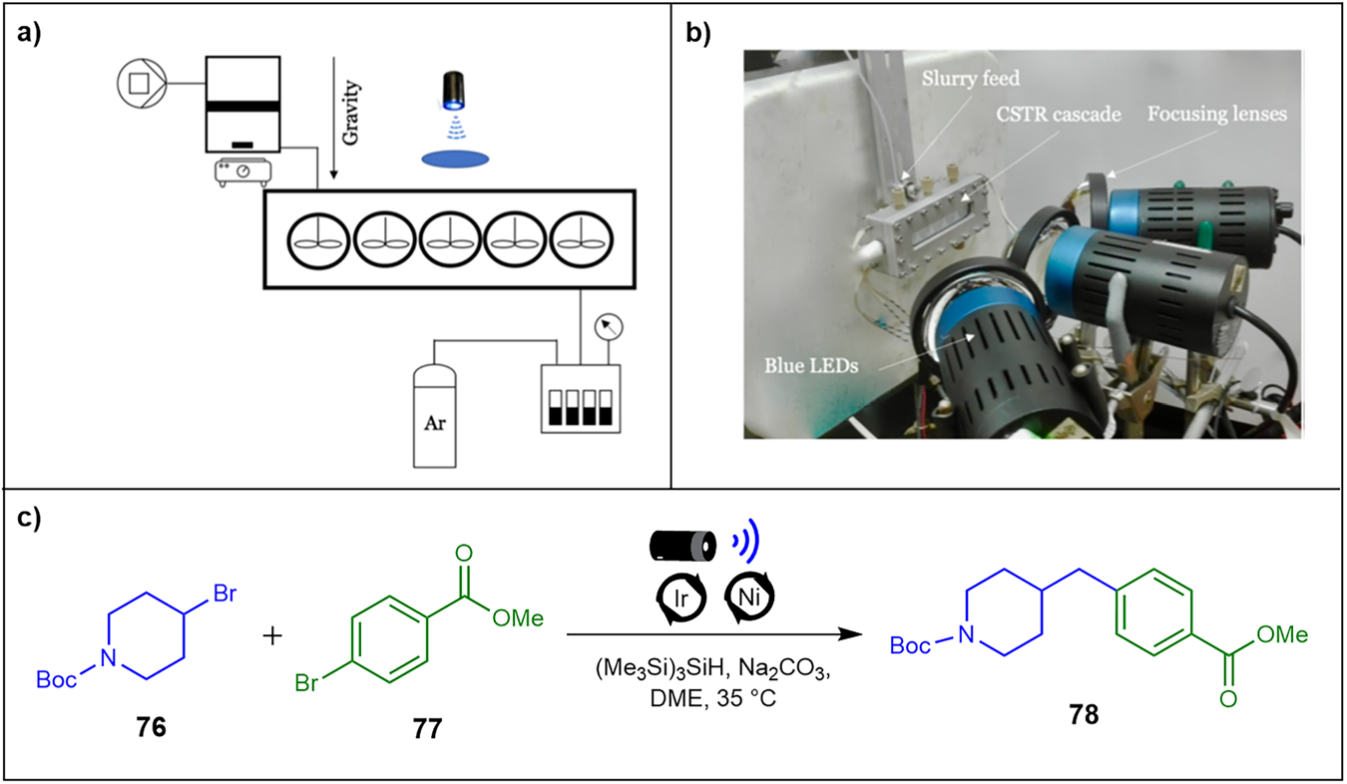
A CSTR platform for heterogeneous visible-light silyl radical-mediated metallaphotoredox cross-coupling reactions. **a** A photo-CSTR platform including the slurry pump CSTR cascade, 3 light-emitting diode light sources (440 nm), focusing lenses, and the pressurised fraction collector. **b** Image of the lamps and reactor. Reproduced from ref. ^136^ with permission from American Chemical Society, copyright [2019]. **c** Silyl radical-mediated metallaphotoredox cross-coupling reaction of **76** and **77**, yielding **78**.

Scaling up photochemical processes in continuous flow synthesis is challenging due to the photon flux limitations of most photochemical reactions, which often involve insoluble starting materials or products that can clog flow reactors. Pomberger and co-workers^136^ addressed this by developing a CSTR platform tailored for heterogeneous visible-light silyl radical-mediated metallaphotoredox cross-coupling reactions, enabling efficient solid handling and improved scalability in continuous flow systems. (Fig. 26). A slurry pump was developed to deliver heterogeneous reaction mixtures into a CSTR cascade for photochemical processes. The pump consisted of a stainless-steel cylinder and an inert hydraulic liquid to ensure controlled and efficient slurry delivery. A dedicated slurry pump addresses non‑Newtonian flow instabilities, while CSTR agitation prevents agglomeration and channel bridging and reduces surface fouling under illumination. A silyl radical-mediated metallaphotoredox cross-coupling reaction of 4-bromotetrahydropyran **76** and methyl-4-bromobenzoate **77** in a photo-CSTR, utilising a photocatalyst, a nickel catalyst, tris(trimethylsilyl)silane (TTMSS), Na₂CO₃, and blue LEDs was demonstrated (Fig. 26). After optimising the slurry pump to minimise clogging and refining reaction conditions, they achieved a 59% yield of product **78** with 80 mg.h^−1^ throughput.

**Fig. 27:**
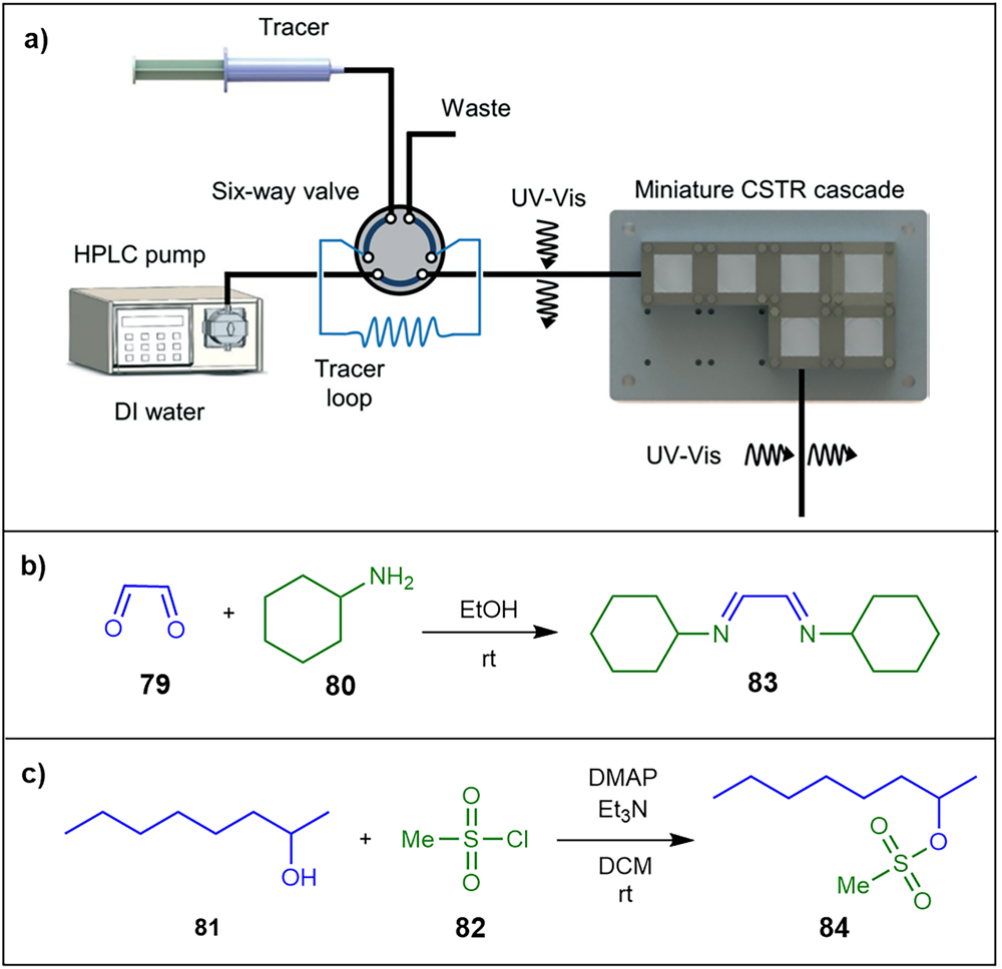
Vertically orientated miniature CSTR cascade for effective solid handling in continuous flow. **a** CSTR system for handling solids formed in a reaction. Reproduced from ref. ^137^ with permission from Creative Commons License, copyright [2016]. **b** Reaction of glyoxal **79** with **80** in the CSTR. **c** Sulfonylation of 2-octanol **81** in the CSTR.

Mo and co-workers^137^ developed a miniature CSTR cascade designed for effective solid handling in continuous flow (Fig. 27a). The single CSTR unit consists of three main components: a polytetrafluoroethylene reactor block, a heat-resistant glass cover, and a stainless-steel cover. The residence time distribution of the cascade was determined using the pulse injection method, and clogging was minimised by operating the CSTRs in vertical mode. Vertical orientation and staged agitation attenuate gravity-driven sedimentation, maintain stable viscosity at modest solids loadings, and avoid wall deposition during extended runs. To evaluate the system’s ability to handle solid formation, the authors conducted two reactions: the reaction of glyoxal **79** with cyclohexylamine **80** and the sulfonylation of 2-octanol **81** with treatment of methanesulfonyl chloride **82** (Fig. 27b). The glyoxal reaction, affording di-imine **83**, demonstrated a 4.4% (w/w) solid handling capacity, achieving full conversion in 15 min using a six-unit CSTR cascade. The sulfonylation reaction, yielding **84**, ran continuously for 8 h without clogging, managing a solid loading of 4.1% (w/w). These results highlight the CSTR cascade’s effectiveness in handling solids in continuous flow processes^137^.

Recent advancements in CSTR technology, as exemplified by the reviewed case studies, demonstrate its effectiveness in handling heterogeneous reactions in continuous flow. Key advantages include robust solid handling, telescoping capabilities and impressive space-time yields. CSTRs offer a powerful tool for modern flow chemists transitioning solid-based reactions from batch to continuous processes, addressing challenges like clogging and inefficient mixing while enabling scalable, high-purity chemicals.

#### Other dynamic mixing reactor systems

In addition to CSTRs, various dynamic mixing systems are employed to manage solids in continuous flow, with a growing range of specialised commercial equipment now available. These include Continuous Oscillatory Baffled Reactors (COBRs), tubular reactors with integrated agitation and spinning disc reactors, which utilise oscillatory, mechanical or centrifugal mixing to prevent solid sedimentation and blockages^138–141^. These advancements enhance the efficiency and scalability of solid handling in pharmaceutical and fine chemical synthesis. COBRs combine plug-flow behaviour with oscillatory mixing to maintain solids in suspension under laminar conditions, while spinning disc and agitated cell reactors deploy high shear and lateral agitation to counter precipitation-induced clogging, suppress anisotropic particle interlocking, stabilise non-Newtonian streams, and reduce adhesion on rotor and wall surfaces^33,138,140,141^. This section discusses various case studies that illustrate the practical implementation of these dynamic mixing technologies, highlighting their effectiveness in managing solids under continuous flow conditions and their impact on process robustness, scalability, and sustainability.

Organolithium reagents, such as *n*-butyllithium (*n*-BuLi) and *n*-hexyllithium (*n*-HexLi), are widely used in synthetic chemistry for deprotonation and halogen-metal exchange reactions, but their application in flow systems is challenging due to precipitation of salt-like by-products such as Li-alkoxides that cause reactor clogging^138,139^. Their high reactivity and pyrophoric nature in air drive their increasing use in flow systems to enhance safety and control^139^. However, these reagents, commonly supplied in hydrocarbon solvents, form insoluble by-products during reactions, leading to reactor blockages in continuous flow systems. This necessitates effective strategies to manage by-product precipitation and prevent reactor blockages.

**Fig. 28:**
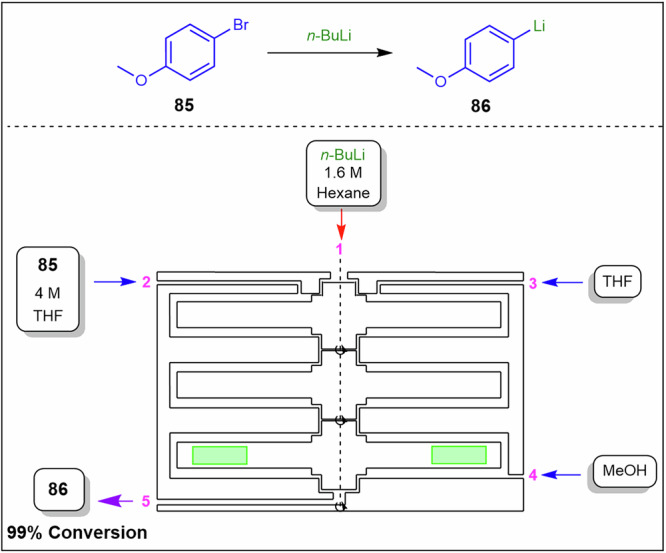
Flow process for the lithiation of **85**. A disc-reactor enabled Br/Li exchange reaction of 4-bromoanisole **85** using *n*-BuLi in the dynamic flow mode^139^.

Wietelmann et al.^139^ demonstrated continuous processing of concentrated organolithiums (*n*-BuLi and *n*-HexLi) using dynamic spinning disc reactors for deprotonation and bromine/lithium exchange reactions. They demonstrated that the presence of hydrocarbon solvents (hexanes) in reaction mixtures in high concentrations may have a detrimental effect on reactions involving organolithium solutions in continuous flow processes, such as reactor blockages and poor productivity^139^. To address this, the authors used a spinning disc continuous flow reactor and reduced hexane content by adding solvents such as THF to *n*-BuLi or *n*-HexLi in deprotonation and bromine/lithium exchange reactions, and prevented precipitation and increased space-time yields. The bromo-lithium exchange reaction of 4 M 4-bromoanisole **85** using *n*-BuLi (5.8 M in hexanes, which was diluted with THF to 1.6 M in the reactor) was performed in a spinning disc reactor at room temperature, affording 99% conversion to lithiated **86** in 5 s residence time without reactor clogging (Fig. 28)^139^. Dilution strategies and thin-film shear on the disc prevent salt precipitation, stabilise viscosity and pressure drop at short residence times, and limit wall adhesion that otherwise drives intermittent fouling.

**Fig. 29:**
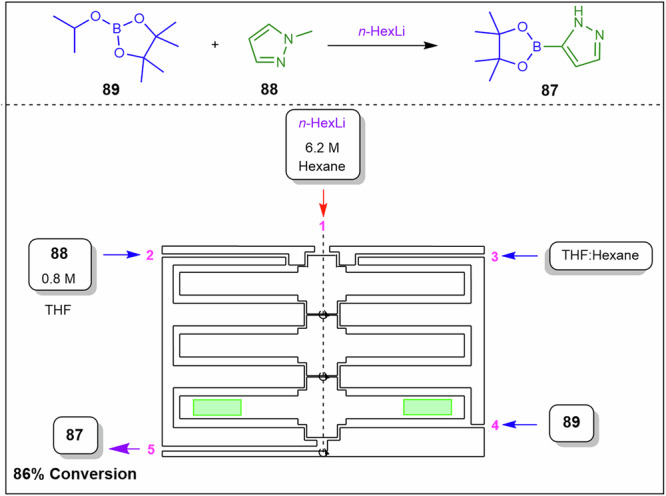
Flow process for the preparation of **87**. A disc-reactor enabled reprotonation–borylation reaction of pyrazole **88** and boronate **89** using *n*-HexLi in the dynamic flow mode^139^.

The authors also demonstrated a deprotonative lithiation reaction, exemplified by the synthesis of a pyrazole boronate **87**, a key intermediate in producing Afuresertib, a GSK AKT inhibitor (Fig. 29)^139^. Pyrazole **88** was reacted with a boronate **89** at 0.8 M concentration using *n*-HexLi (6.2 M) in a spinning disc reactor at −20 °C, achieving 86% conversion in 18 s without reactor clogging. The spinning disc reactor’s robust design tolerated salt precipitation, ensured excellent mass transfer, and established a foundation for efficient handling of organolithium reagents in continuous flow, enabling scalable production of pharmaceutical intermediates, agrochemicals, and specialty chemicals.

Diazotization is a crucial process in various fields and serves as a source of important diazonium intermediates. However, similarly to organometallic reagents, diazo compounds are highly reactive, unstable, explosive, and prone to rapid decomposition, making their synthesis using classical methods unsafe. To address these safety concerns, many researchers have turned to continuous flow techniques for generating diazonium intermediates. A major challenge in continuous flow systems is the formation of diazonium salts, which can cause pressure drops due to clogging.

**Fig. 30:**
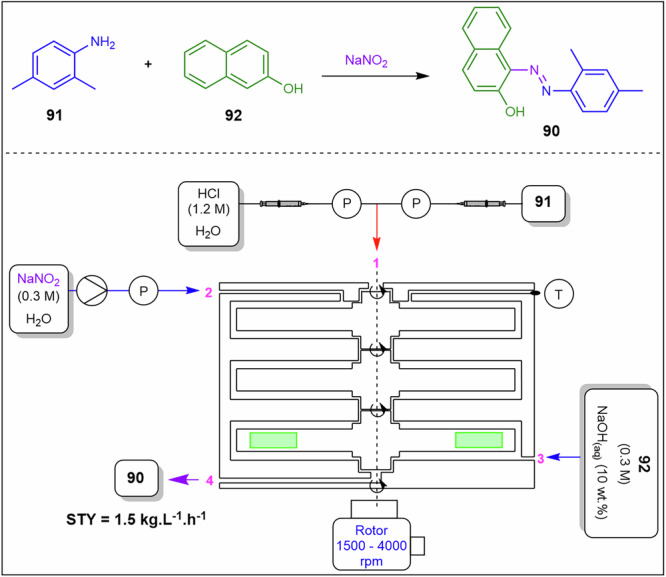
Continuous flow process for the preparation of Sudan II dye **90**. Flow system for the synthesis of Sudan II dye **90** via diazonium salts formation in situ and subsequent coupling in a spinning disc reactor.

To overcome this, Polterauer et al.^142^ employed a spinning disc reactor to produce diazonium salts in situ and subsequently couple them (Fig. 30). As a demonstration, they synthesised the Sudan II dye **90** from 2,4-dimethylaniline **91** by first forming the corresponding diazonium salt, which was subsequently coupled with 2-naphthol **92** as a model reaction. They achieved high conversions, successfully managed the solid byproduct, and used water as the reaction solvent. Disc‑generated films mitigate rapid precipitation and aggregation of diazonium salts, stabilise hydrodynamics in exothermic regimes that influence viscosity, and reduce deposit formation on wetted surfaces. In their stoichiometric optimisations, the best results were obtained using 1.2 eq. of **91**, 2.4 eq. of HCl, 1.2 eq. of NaNO₂, and 1.0 eq. of 2-naphthol **92**. After comprehensive process optimisation involving temperature, rotation speed, and residence time, the system produced 28.5 g.h^−1^ (684 g.day^−1^) in a 19 mL reactor, corresponding to a space-time yield of 1.5 kg.L^−1^.h^−1^. The product was easily isolated by filtration, washing, and drying, yielding a highly pure dye.

The Coflore® agitated cell reactor (ACR) represents a significant evolution over the conventional CSTR. Unlike a classical CSTR, which consists of a single well-mixed vessel, the Coflore ACR is composed of ten discrete reactor cells arranged in series, effectively functioning as a cascade of ten CSTRs^143,144^. This configuration allows the system to approximate plug-flow reactor behaviour while retaining the advantages of continuous stirring. To mitigate common issues associated with traditional single-tube CSTRs, such as uncontrolled bypassing and back-mixing, the Coflore ACR incorporates interstage channels between each reactor cell. These channels ensure more uniform residence time distribution and minimize short-circuiting of reactants, thereby enhancing reaction efficiency and selectivity. Furthermore, dynamic mixing within each reactor cell is enhanced through lateral agitation of the entire reactor cell block, which can be controlled in the frequency range of 2–6 Hz. This agitation promotes homogeneous reactant distribution, improves mass and heat transfer, and prevents the formation of concentration gradients within individual cells, ensuring consistent reaction conditions throughout the reactor^143,144^. Compartmentalised flow approximates plug‑flow behaviour while maintaining agitation that controls slurry rheology, breaks particle networks to prevent bridging, and reduces wall adhesion by minimising stagnant zones.

**Fig. 31:**
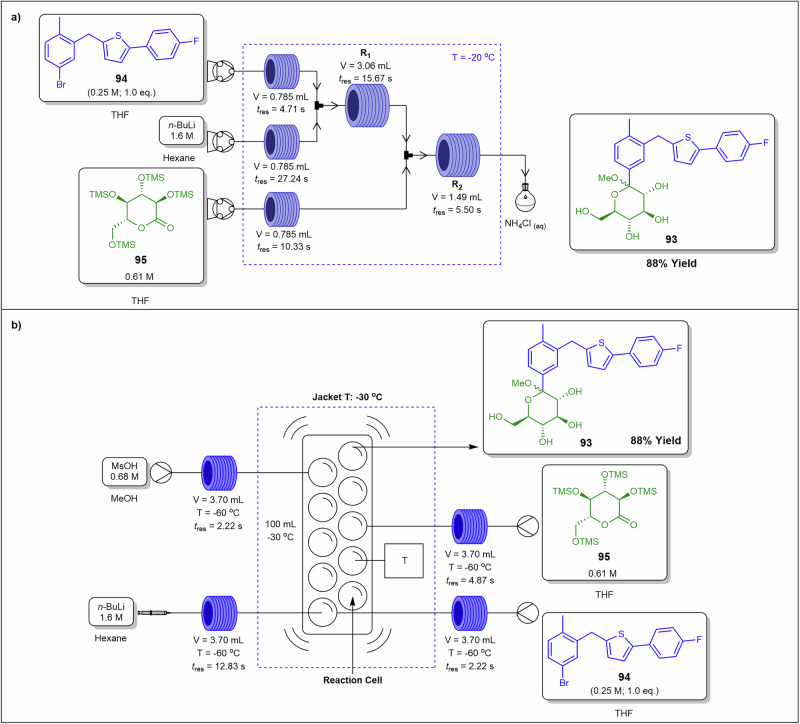
Continuous flows synthesis of canagliflozin intermediate **93**. **a** Continuous flow synthesis of canagliflozin intermediate **93** using a plug-flow reactor. **b** Continuous flow synthesis of canagliflozin intermediate **93** using a Coflore ACR.

Yano et al.^145^ compared batch, classic continuous plug flow reactor, and the Coflore ACR in the synthesis of compound **93**, which is a key intermediate for the synthesis of Canagliflozin (Fig. 31). The ACR demonstrates the efficient handling of in situ solid side-product, LiOH formation by preventing settling, stabilising viscosity and pressure profiles, and limiting adhesion that drives run-to-run pressure drift in single-tube systems. The batch process afforded **93** in 50% and 8% yields at −78 and −40 °C, respectively. Notably, the low yields were due to unstable *n*-BuLi and competing side reactions. Furthermore, the authors performed a continuous plug flow synthesis of **93** and optimised the process with the use of a three-pump setup consisting of an initial lithiation of 2-(5-bromo-2-methylbenzyl)-5-(4-fluorophenyl)thiophene **94** with *n*-BuLi, followed by the introduction of TMS protected **95** before being treated with ammonium chloride affording **93** in 88% yield (Fig. 31a). The Coflore ACR achieved an exceptionally fast residence time of 3.5 min, efficiently managed the solids formed during the reaction, and produced 88% of the desired product **93** which was comparable to the plug-flow conditions (Fig. 31b).

**Fig. 32:**
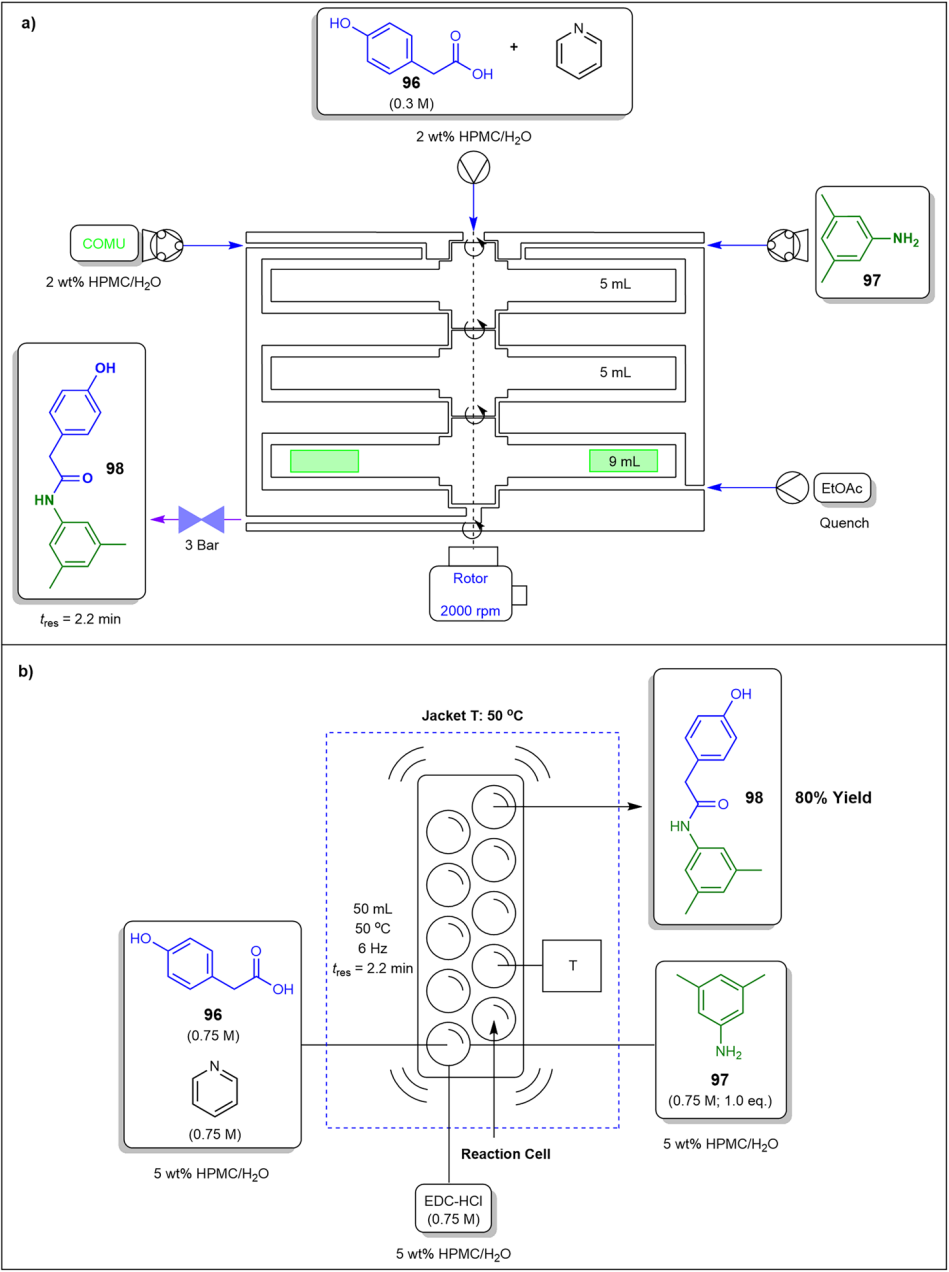
Continuous flow amidation under aqueous conditions using EDC for **98** synthesis. **a** Continuous flow amidation using SDR for the preparation of **98**. **b** Continuous flow amidation using Coflore ACR for the preparation of **98**.

Rajendran et al.^146^ employed both the SpinPro R2 spinning disk reactor (SDR) and the Coflore ACR to facilitate the synthesis of an amide functional group from carboxylic acids and amines in aqueous media (Fig. 32). Both systems involved slurries, which were delivered via peristaltic pumps from a vigorously stirred vessel.

During reaction optimisation, 1-Ethyl-3-(3-dimethylaminopropyl)carbodiimide (EDC) was identified as the most effective coupling reagent due to its facile removal during aqueous work-up. A recurring challenge with the SDR was intermittent pressure drops caused by solids adhering to the rotor. This issue was effectively mitigated using the Coflore ACR, which allowed extended continuous operation without pressure fluctuations. Under optimised conditions, 2-(4-hydroxyphenyl)acetic acid **96** was successfully coupled with 3,5-dimethylaniline **97** affording **98** with an isolated yield of 80%, a throughput of 1.5 kg.day^−1^, and a STY of 1.4 kg.L⁻¹.h⁻¹ (Fig. 32). The ACR’s lateral agitation suppresses wall fouling and sedimentation while keeping slurry viscosity within a stable operating window.

**Fig. 33:**
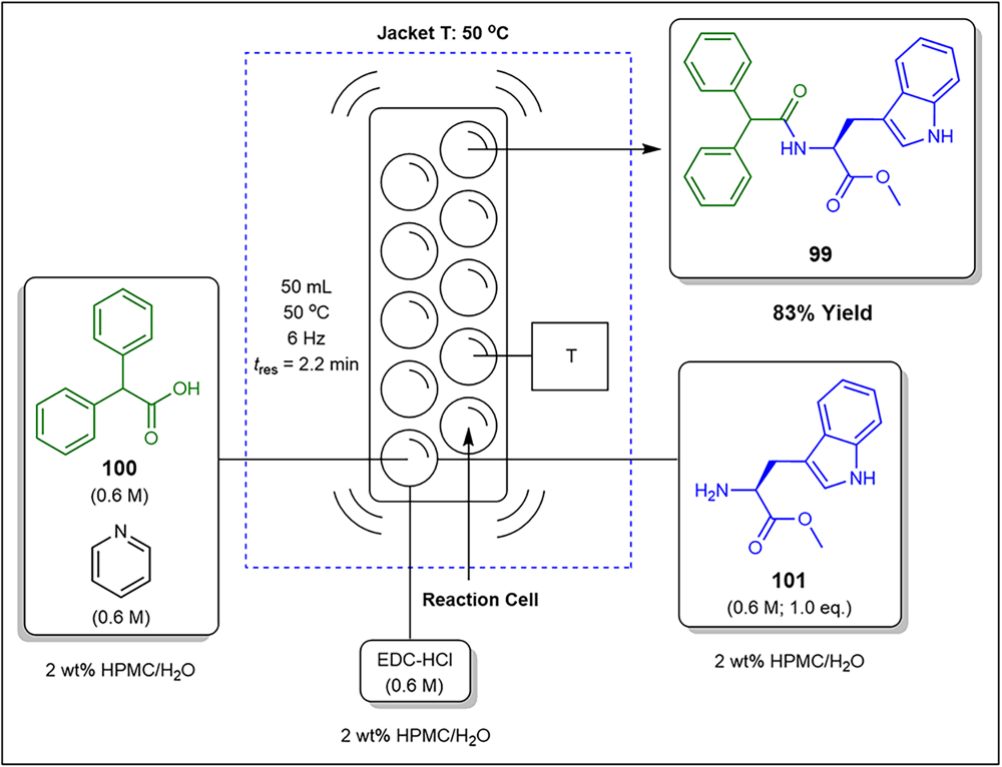
Flow process for the preparation of **99**. Continuous flow amidation under aqueous conditions using a Coflore ACR for **99** synthesis.

Importantly, this continuous aqueous process avoided the use of organic solvents, required no excess reagents, and sustained a 3-h continuous run with the slurry feedstock without clogging, maintaining an average yield of 80%. A similar methodology was applied to the synthesis of amide-containing **99** from carboxylic acid **100** and amine **101**. Initially validated in batch, the process was subsequently scaled to achieve a productivity of 2.0 kg.day^−1^ (Fig. 33), exhibiting comparable operational benefits. Furthermore, green chemistry metrics favoured this approach, demonstrating a low E-factor and process mass intensity, minimal waste generation, and facile scale-up potential.

Yao et al.^147^ explored the translation of the batch synthesis of **102** from the treatment of 2,2′-methylene-bis(4,6-di-*tert*-butylphenol) **103** with POCl₃ into a continuous process. Initially, a PFA coil reactor was employed with various solvents to maintain solution homogeneity; however, clogging was observed. Attempts to mitigate this issue using ultrasonic irradiation were unsuccessful, likely due to the effects of external mechanical energy. Subsequently, a continuous vibrating coil reactor (CVCR) a recently developed system with operational principles similar to a CSTR, was tested. Using the CVCR, optimal conditions of 4 min residence time, 1.1 eq. of POCl₃, 3.0 eq. of triethylamine at 30 °C yielded 75.9% of **102**. Nevertheless, clogging recurred within 30 min of operation.

**Fig. 34:**
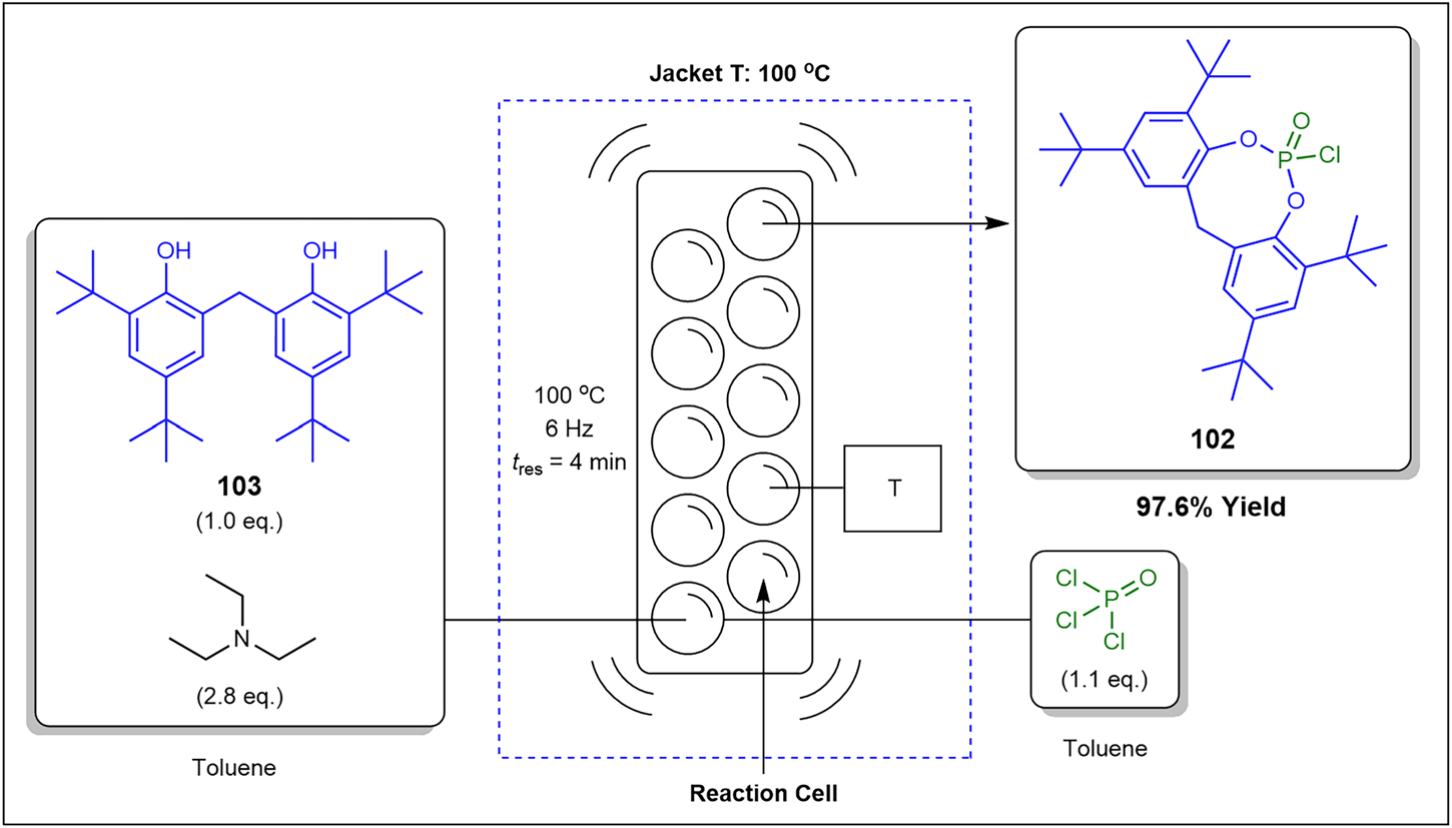
Flow process for the preparation of **102**. Active mixing enabled continuous flow synthesis of **102** under continuous flow conditions using a Coflore ACR.

Ultimately, the authors employed a Coflore ACR for the preparation of **102** (Fig. 34). The ACR enabled a straightforward setup, short residence time, and easy scale-up. Key reaction parameters, including shaking frequency, temperature, and stoichiometry of POCl₃ and triethylamine, were optimised. The reactor effectively handled the solid product over several hours, achieving an excellent yield of up to 98% with a 4-min residence time at 100 °C. Persistent clogging in a PFA coil reactor implicates precipitation and adhesion; the ACR’s compartmental agitation and short inter-stage paths prevent agglomeration and settling, stabilising pressure and residence time despite solid formation.

**Fig. 35:**
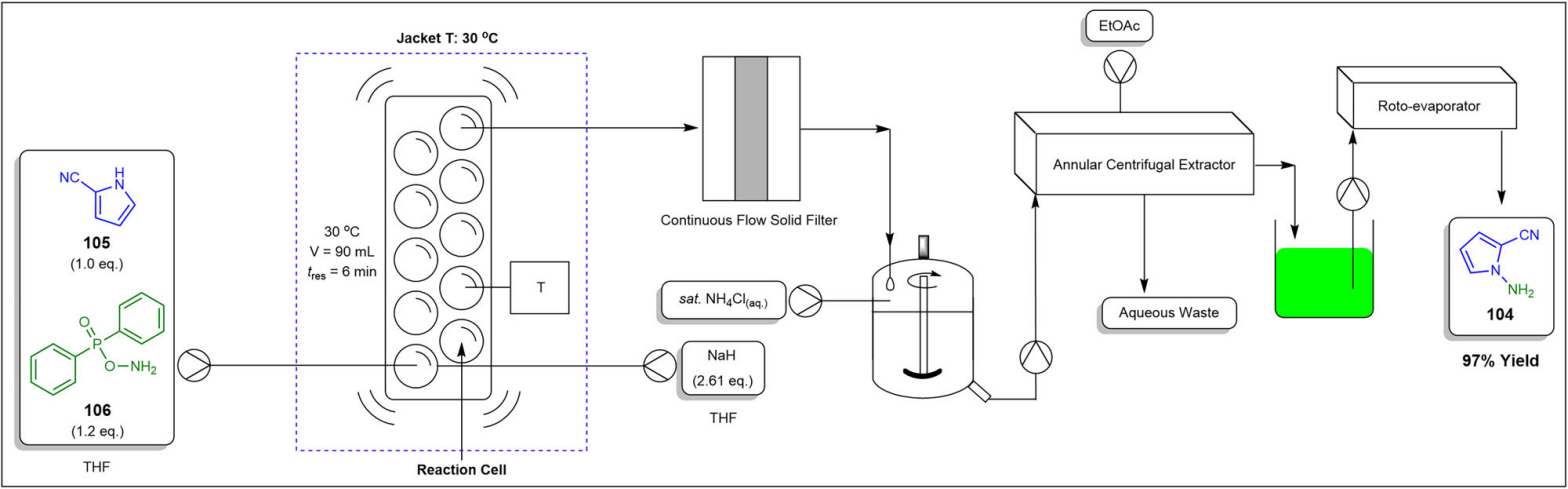
Flow process for the preparation of nucleobase unit **104**. Continuous flow slurry synthesis of nucleobase unit **104** of the antiviral drug remdesivir using a Coflore ACR.

The use of continuous ACRs is gaining popularity for the continuous handling of solid slurries. Guo et al.^148^ employed an ACR in the synthesis of the nucleobase unit **104** of the antiviral drug remdesivir (Fig. 35). The ACR was chosen because NaH and Ph₂P(O)ONH₂ are insoluble in THF, making conventional batch methods challenging. After compressive optimisation, 2-cyanopyrrole **105** and diphenyl containing **106** were successfully converted to **104**, with the authors having achieved a 97% isolated yield with a 6 min residence time. Moreover, reactions run for up to 10 h proceeded without clogging, highlighting the ACR’s effectiveness in managing solid-containing reactions. Active mixing in the ACR controls insoluble base and reagent particles, maintains uniform viscosity and throughput over long runs, and reduces adhesion on surfaces.

**Fig. 36:**
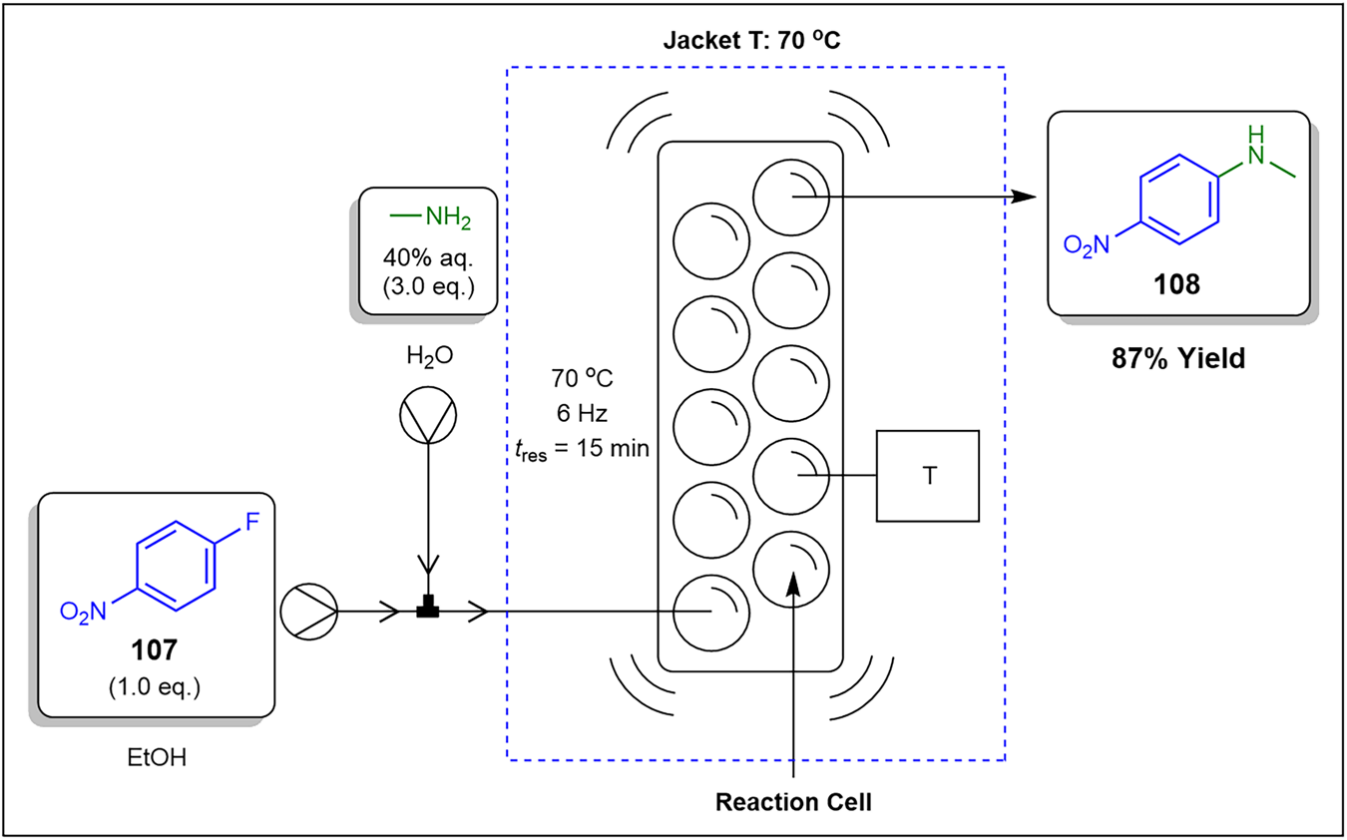
Continuous flow process for the preparation of **108**. Continuous flow amination of 4-fluoronitrobenzene **107** using a Coflore ACR.

Furthermore, Luo and co-workers^149^ used an ACR system to manage solids in the amination of 4-fluoronitrobenzene **107** by using methylamine aqueous solution as the amination reagent (Fig. 36). The reaction proceeded efficiently, yielding the desired product **108** in high yield (> 90%) and excellent purity within 15 min at 70 °C. Their novel, facile, and environmentally friendly continuous method also produces a uniform particle size distribution, enhancing the practicality and economic viability of the procedure. Uniform particle sizing and agitation prevent sedimentation, stabilise viscosity at short residence times, and avoid wall fouling that typically escalates pressure in single-tube reactors.

**Fig. 37:**
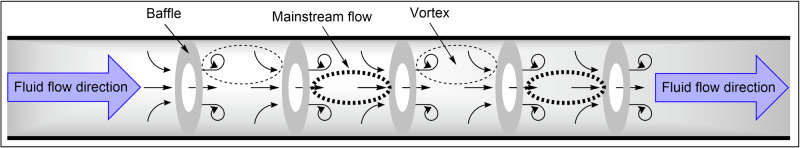
Cross sectioned fluid flow in a continuous oscillatory baffled reactor (COBR). Illustration highlighting the inner characteristics of a COBR, containing segmented regimes composed of a baffle, the mainstream flow and vortex formations because of the internal structural design.

Continuous oscillatory baffled reactors (COBRs), also known as oscillatory flow reactors (OFRs), are a specialised class of dynamic mixing systems that enable efficient solids handling under continuous flow conditions^33,150^. Unlike conventional tubular reactors that rely on high linear velocities to achieve mixing, COBRs superimpose oscillatory motion on a net forward flow within a baffled tube (Fig. 37). This oscillatory regime generates periodic vortices between baffles, creating uniform mixing zones that maintain solids in suspension even at low net flow rates^151–153^. By decoupling mixing intensity from bulk flow velocity, COBRs achieve high mass and heat transfer efficiency under laminar conditions, reducing shear stress and preserving particle integrity.

The hydrodynamic characteristics of COBRs combine plug-flow behaviour with enhanced radial mixing, delivering controlled residence time distribution while mitigating common solids-related challenges such as sedimentation, agglomeration, and fouling^151,153^. These features make COBRs particularly attractive for processes involving fragile crystals, heterogeneous catalysts, or precipitating species, where conventional high-shear or high-velocity systems may compromise product quality or reactor operability.

COBRs have demonstrated significant advantages in continuous crystallization, precipitation, and slurry-phase reactions, offering superior control over particle size distribution and reducing fouling compared to stirred tanks^33,154,155^. Their ability to maintain suspension stability under low shear conditions ensures uniform exposure of solids to reaction environments, improving process robustness and scalability. Operational success depends on careful tuning of oscillation frequency and amplitude relative to baffle geometry and slurry rheology, while pumping strategies must accommodate oscillatory flow, often favouring diaphragm or peristaltic pumps for solids-containing streams^151,152^. Scale-up typically relies on numbering-up rather than increasing diameter to preserve oscillatory Reynolds numbers and vortex dynamics^156^.

By integrating oscillatory mixing with continuous flow, COBRs complement other dynamic reactor technologies and provide a scalable, solids-compatible solution for sustainable chemical manufacturing. Although COBRs are employed in both upstream and downstream processes, this review focuses exclusively on upstream case studies to demonstrate their role in managing solids.

**Fig. 38:**
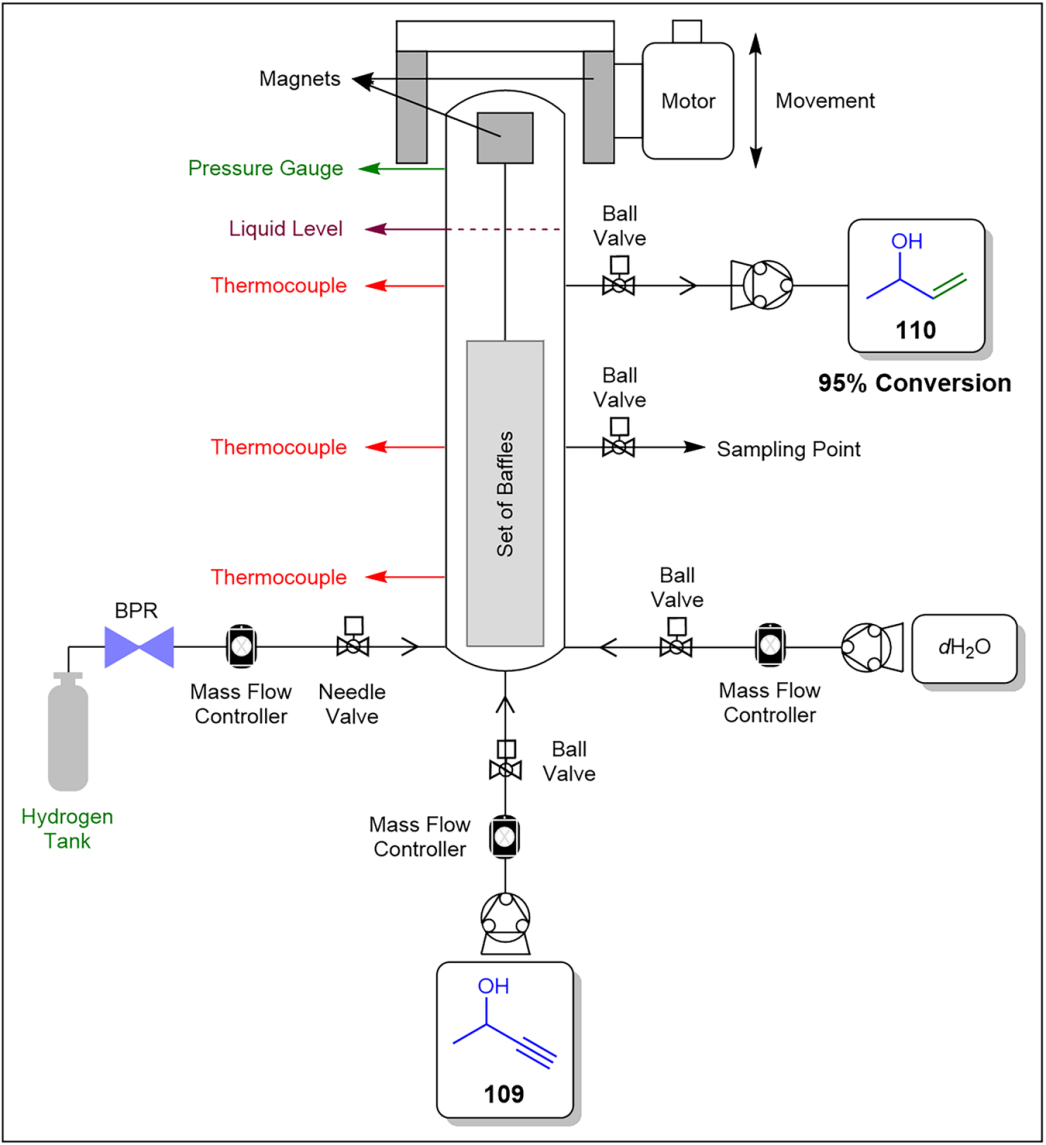
Continuous flow process for the preparation of **110**. Selective hydrogenation of hydroxy-containing alkyne **109** to **110** in a COBR.

Navarro-Fuentes et al.^157^ reported the first successful extended application of a COBR for multiphase catalytic hydrogenation of 3-butyn-2-ol **109** over Pd/Al₂O₃ under continuous flow conditions (Fig. 38). The reactor exhibited catalyst stability and prevented attrition throughout 12 h of operation, achieving 95% conversion and 97% selectivity for 3-buten-2-ol **110**. Catalyst deactivation was negligible for the first 9 h, with a rate of 0.03% per h, thereafter, projecting 17 days (2040 cycles) to reach 50% of target conversion, which significantly outperformed batch operation that exhibited 1.5% deactivation per cycle and reached 50% loss after 32 cycles.

The COBR’s oscillatory mixing regime ensured uniform suspension of catalyst particles, mitigating sedimentation and fouling while enhancing gas–liquid–solid contact. This design enabled rapid attainment of steady-state conditions within one residence time (~ 12 min), allowing continuous screening of operational parameters such as oscillatory velocity, residence time, and pressure. Increased oscillatory velocity improved conversion by reducing bubble size, increasing gas hold-up, and prolonging bubble residence time, confirming mass-transfer limitations as the controlling factor.

**Fig. 39:**
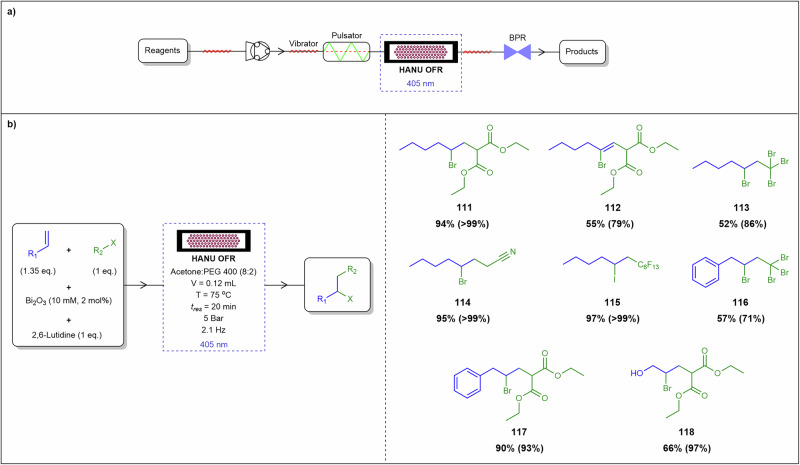
Handling solids in atom transfer radical addition reactions. **a** Oscillatory flow reactor setup **b** atom transfer radical addition reactions affording **111**–**118** using an Oscillatory flow reactor^158^.

Bianchi and co-workers^158^ developed a continuous flow protocol for atom transfer radical addition (ATRA) using an oscillatory flow reactor to overcome challenges in handling dense solid photocatalysts under photochemical conditions (Fig. 39). The process employed Bi₂O₃ as a sustainable photocatalyst with visible-light irradiation and a green solvent mixture of acetone and PEG 400 to maintain suspension stability. Oscillatory mixing combined with static elements ensured uniform dispersion of Bi₂O₃ and prevented clogging during extended operation.

Under optimised conditions (2 mol% Bi₂O₃, 75 °C, 15–20 min residence time), the system achieved 99% conversion and 94% yield with a space-time yield of 599 g L⁻¹ h⁻¹. A four-hour continuous run processed 250 mL without settling, and Bi₂O₃ was successfully recycled over three cycles. The method demonstrated excellent operational stability, sustainability, and broad substrate scope, delivering multigram-scale products **111**–**118** in moderate to high yields.

**Fig. 40:**
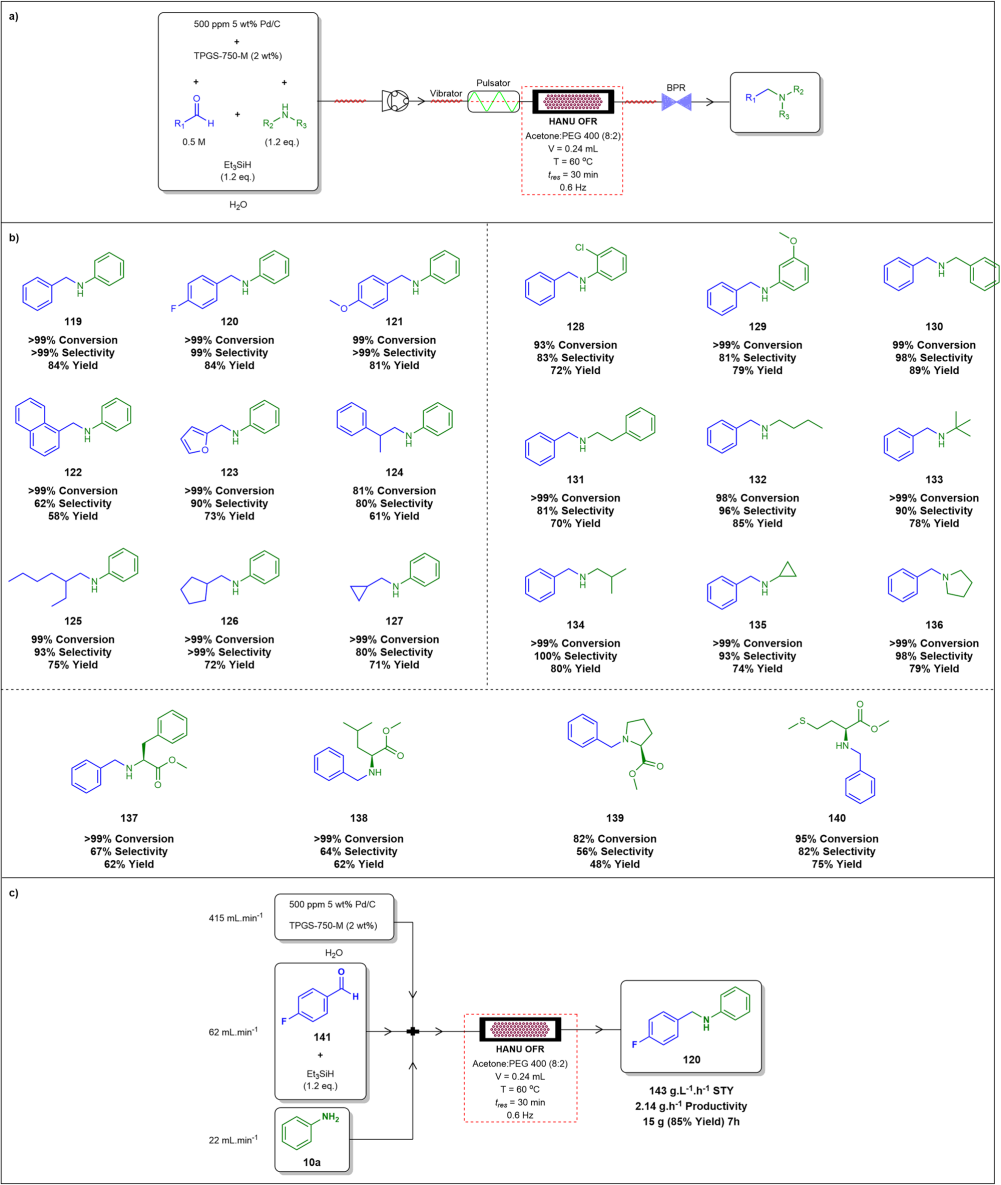
Heterogeneous catalytic reductive aminations affording **119**–**140**. **a** Oscillatory flow reactor setup. **b** Substrate scope for reductive aminations in OFR. **c** Long run experiment under optimised flow conditions for compound **120**.

Wernik et al.^150^ demonstrated a continuous flow process for heterogeneous catalytic reductive aminations under aqueous micellar conditions using an oscillatory plug flow reactor (Fig. 40a). The system employed ppm levels of Pd/C with Et₃SiH as reductant and TPGS-750-M as surfactant, enabling efficient micellar catalysis in water. Oscillatory mixing combined with static elements ensured stable suspension of solids and enhanced mass transfer, overcoming limitations of batch scalability and clogging risks.

Under optimized conditions (500 ppm Pd/C, 60 °C, 30 min residence time), the process achieved quantitative conversion and selectivity across a broad substrate scope affording **119**–**136** (Fig. 40b), including amino acid derivatives **137**–**140**. A seven-hour long-run involving aniline **10a** and 4-fluorobenzaldehyde **141** produced 15 g of product **120** with an 85% yield and a space-time yield of 143 g L⁻¹ h⁻¹ (Fig. 40c). Catalyst and surfactant recycling over five cycles maintained full activity, and the process delivered excellent sustainability metrics with an E-factor of 1.2.

As demonstrated by the reviewed case studies, COBRs provide an effective and scalable solution for solids handling in continuous flow by combining controlled mixing, stable suspension, and enhanced mass and heat transfer. Their versatility has been proven in many chemical transformations including hydrogenation, photochemical ATRA, and reductive amination processes, delivering high productivity and sustainability. For further insights into COBR design principles, operational parameters, and broader applications such as crystallization, precipitation, and slurry-phase reactions, readers are referred to the comprehensive review by Bianchi et al.^33^, which offers an in-depth discussion of this technology within modern chemical manufacturing frameworks.

Evidently, the case studies in this section have demonstrated that dynamically mixing continuous flow reactor systems are increasingly valuable for reactions that challenge classical flow configurations because they enhance mixing regimes to overcome limitations such as poor mass transfer, heterogeneous phase handling, and inefficient kinetics. A key advantage is their ability to manage solids, which remains a major barrier in continuous processing. The spinning disk reactor forms thin liquid films under intense shear to enable efficient heat and mass transfer while minimizing fouling, the agitated cell reactor uses compartmentalized design and lateral agitation to ensure stable slurry handling and prevent sedimentation, and COBRs maintain solids suspension under low shear conditions while combining controlled residence time with gentle mixing to preserve particle integrity. Together, these technologies demonstrate how dynamic mixing strategies provide robust solutions for processes prone to clogging, sedimentation, or poor performance in conventional systems.

Continued progress in the development of solid-tolerant reactor architectures will facilitate scalable access to transformations that have historically been considered intractable, while simultaneously supporting the broader objectives of sustainable and resource-efficient chemical production. By combining innovative reactor designs with complementary chemical strategies, the field is moving toward a more resilient and versatile paradigm for handling solids in continuous flow processes.

### Chemical reaction modifications

Building on the reactor-based strategies discussed in the previous sections, this part of the review examines chemical modifications that complement engineering solutions for effective solid handling under continuous flow conditions. While reactor design innovations have significantly improved the ability to process solids, chemical adjustments remain a critical dimension in overcoming persistent challenges such as maintaining flow stability and preventing blockages. These challenges are particularly pronounced when dealing with solid reagents, insoluble intermediates, or by-products that can compromise process reliability and scalability. Reaction modifications target precipitation pathways directly, thereby lowering solids-driven viscosity growth, reducing adhesion and fouling in channels, and enabling the use of precision pumps in place of slurry delivery.

A growing body of literature demonstrates that strategic chemical modifications can provide robust and versatile solutions to these issues^25,27,159^. Such modifications encompass a range of approaches, including adjusting reagent concentrations and reaction temperatures, substituting inorganic bases with organic alternatives, changing solvent systems to improve solubility profiles, employing alternative reagents with enhanced compatibility, and even redesigning synthetic routes to minimise solid formation^25,27,159^. These interventions not only mitigate operational risks but also enhance the overall compatibility of solid-handling processes with continuous flow systems. By improving efficiency, scalability, and process robustness, chemical modifications serve as an essential complement to engineering strategies, reinforcing the integrated approach required for modern continuous manufacturing. This section discusses various chemical reaction modifications strategies in solid handling. This section critically examines these chemical modification strategies, highlighting their principles, practical applications, and implications for sustainable and scalable process development.

Adjusting reagent concentrations is a widely reported strategy to mitigate solid formation in flow systems^159^. This approach is particularly effective in reactions generating insoluble by-products, where precise control of concentration ensures solubility without compromising reaction kinetics. The substitution of inorganic bases, such as sodium hydroxide, with organic bases such as triethylamine and (1,8-diazabicyclo[5.4.0]undec-7-ene) DBU has been highlighted as another effective modification^160^. Organic bases often improve solubility in organic solvents, minimising solid precipitation during reactions.

Solvent selection plays a critical role in solid management, as solvents influence the solubility of reagents, products, and by-products. Solvent mixtures tailored to specific reaction conditions have been shown to balance solubility and reaction efficiency, further enhancing process robustness^25^. The adoption of alternative reagents is another viable strategy to circumvent solid-handling issues. Such substitutions not only reduce the risk of clogging but may also improve reaction control under continuous flow conditions. Developing alternative synthetic routes offers a more fundamental solution to solid-handling challenges^160^. By redesigning reaction pathways to avoid the formation of insoluble species, researchers can achieve seamless integration with continuous flow systems. These chemical modification strategies, supported by extensive literature, underscore the adaptability of continuous flow systems in handling solids^160^.

**Fig. 41:**
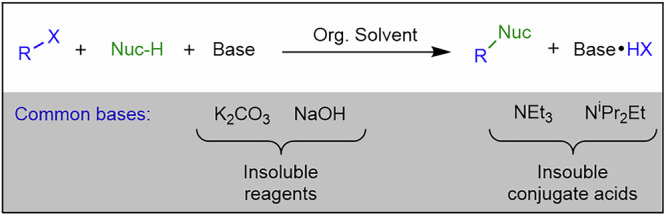
General reaction scheme for insoluble base·HX salt formation in typical substitution reactions^25^. Reaction scheme displaying the typical reaction products obtained during substitution reactions, where Nuc-H is the protonated nucleophile, R-X is any organic moiety bonded with a good leaving group (generally halogens) and R-Nuc is the desired substituted product.

Acylations, arylations, and alkylations are fundamental transformations in synthetic chemistry, particularly in the pharmaceutical and specialty chemical industries. These reactions typically involve a general mechanism where a stoichiometric base scavenges the acid by-product, forming an insoluble base·HX salt (Fig. 41)^25^. In batch processing, these low-solubility salts, which precipitate in common organic solvents, do not cause serious processing challenges. However, in continuous flow systems, the precipitation of base·HX salts within reactor channels often leads to blockages, disrupting flow and reducing process efficiency^24–29^. To mitigate clogging, large solvent volumes are often used to maintain low reagent concentrations, thus preventing precipitation^25^. Unfortunately, this approach is neither cost-effective nor environmentally sustainable, as it increases solvent costs and generates significant waste, making it industrially impractical. Recycling these solvents may be an option, but although this reduces the solvent burden, it concurrently increases the energy burden, and one needs to carefully weigh up if a recycling strategy is both cost-effective and/or sustainable. Strategies to address these challenges, such as alternative base selection or solvent optimisation, are critical for enhancing the efficiency and sustainability of flow-based processes.

Kashani et al.^25^ developed an effective strategy to manage insoluble base·HX salt by-products in continuous flow substitution reactions. By employing acid-scavenging organic bases that form low- to moderate-melting ionic liquids upon protonation, they prevented precipitation and reactor clogging. They validated this approach through acylation, arylation (S_N_Ar), alkylation (S_N_2), and silylation reactions, focusing on bases such as DBU, tributylamine, and *N*-butylimidazole, which produce ionic liquid by-products to enable precipitate-free reactions with N, O, and S nucleophiles.

**Fig. 42:**
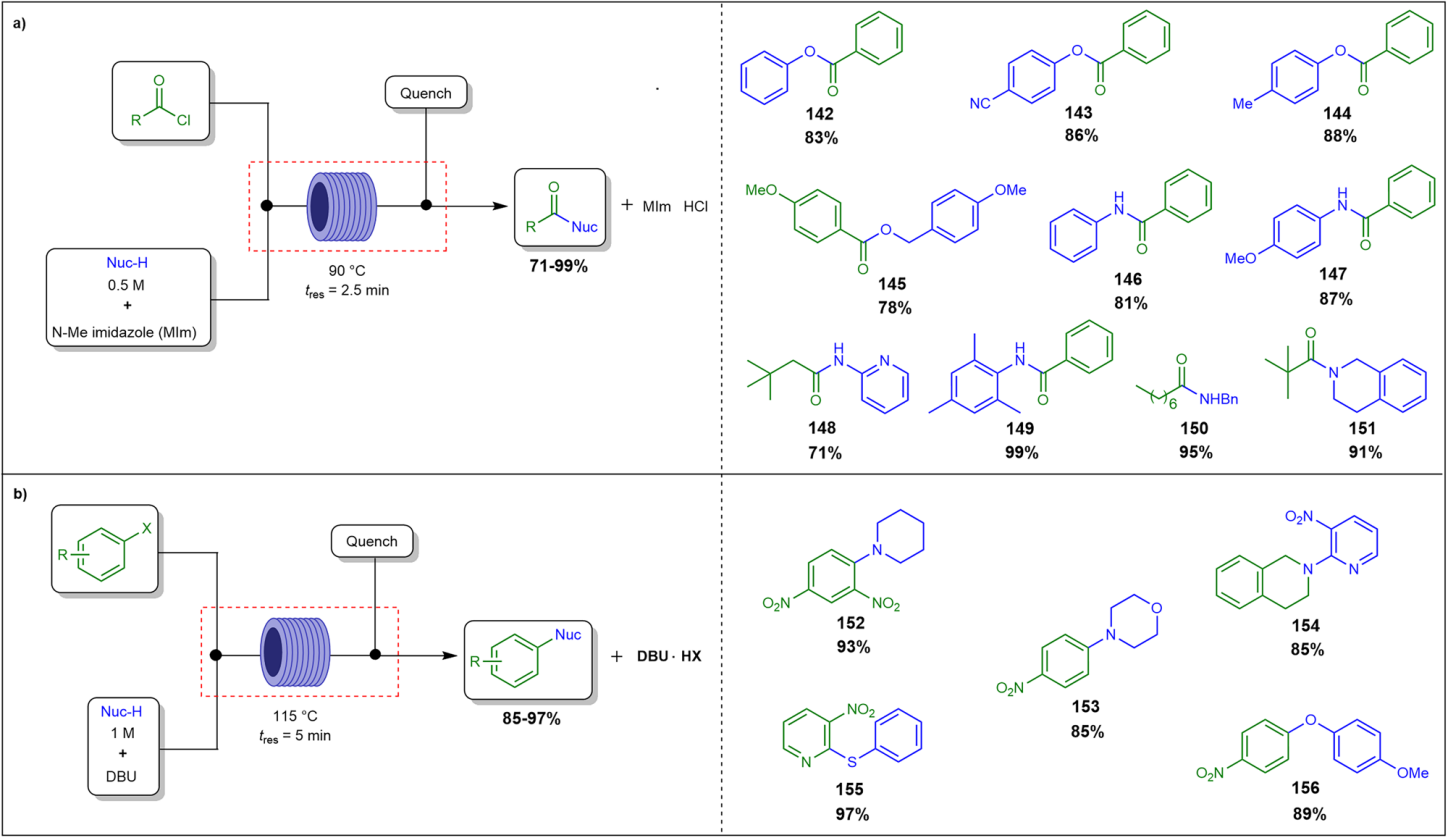
Managing solids in continuous flow systems by using acid-scavenging organic bases. **a** Continuous flow acylation reactions without clogging. **b** Continuous flow of S_N_Ar reaction without clogging.

Continuous flow acylation reactions at 0.5 M concentration in toluene and THF at 90 °C, using *N*-methylimidazole as the base, produced various esters **142**–**145** and amides **146**–**151** in high yields (85–97%) within 2.5 min, with no reactor clogging (Fig. 42a)^25^. To demonstrate that ionic liquid-forming acid scavengers *N*-methylimidazole and DBU prevent continuous flow reactor clogging in S_N_Ar reactions, experiments were performed at 1 M concentration in NMP at 115 °C for 5 min using diverse nucleophilic and electrophilic partners affording products **152**–**156** with a range of 85–97% yields (Fig. 42b)^25^. DBU and *N*-methylimidazole effectively inhibited amine nucleophile protonation and precipitation, ensuring clog-free operation.

**Fig. 43:**
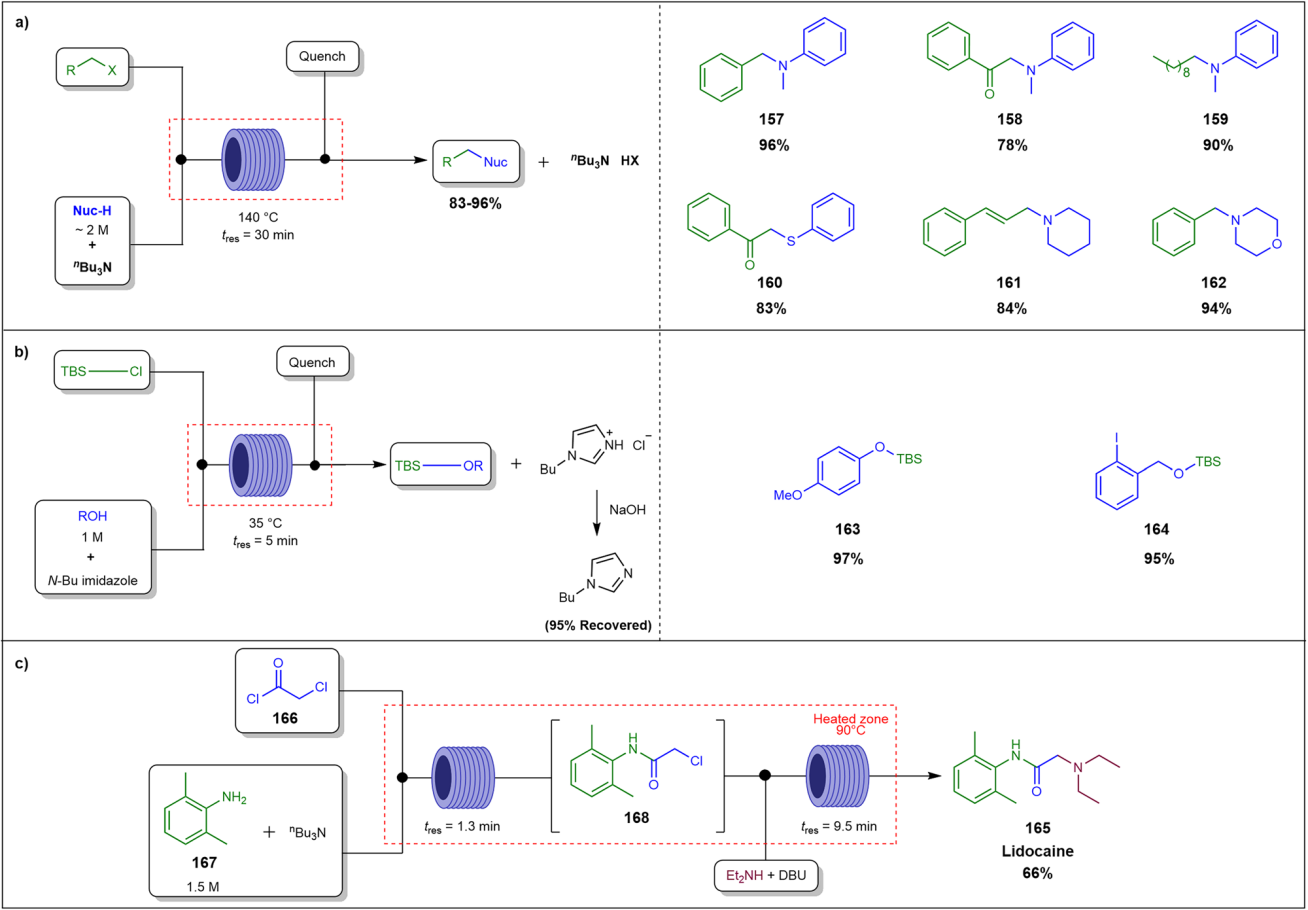
Managing solids in continuous flow systems by using acid-scavenging organic bases. **a** Continuous flow S_N_2 reactions without reactor clogging. **b** Continuous flow silylation reactions without reactor clogging. **c** Telescoped continuous flow synthesis of lidocaine **165** without reactor clogging using Bu_3_N and DBU ionic liquids^25^.

DBU and ^*n*^Bu_3_N were effectively used as ionic liquid-forming acid scavengers in S_N_2 reactions at ~2 M concentration in NMP at 140 °C with a 30-min residence time (Fig. 43a)^25^. Employing various nucleophilic and electrophilic partners, these bases prevented reactor clogging, yielding products **157**–**162** in 78–96% yield. In silylation reactions, silyl ethers **163** and **164** were successfully synthesised in high yields (95–97%) using TBSCl and alcohols at 1 M concentration in NMP at 35 °C, with *N*-butylimidazole as the base, preventing reactor clogging (Fig. 43b)^25^. Due to the high cost of *N*-butylimidazole, it was recovered at 95% efficiency using NaOH for reuse.

After successfully demonstrating an effective strategy to manage insoluble base·HX salts using acid-scavenging organic bases that form low- to moderate-melting ionic liquids in various substitution reactions with diverse substrates, Kashani et al.^25^ applied this approach to a multistep synthesis of the local anaesthetic lidocaine **165** (Fig. 43c). By employing ionic liquid-forming acid scavengers in telescoped flow procedures, they conducted an initial acylation reaction between chloroacetyl chloride **166** and 2,6-dimethylaniline **167**, generating intermediate **168** in situ. The freshly prepared **168** is then subjected to a subsequent alkylation reaction with the treatment of diethylamine, achieving an average yield of 66% with a total residence time of 10.8 min, being comparable to yields reported in the literature^25^. By suppressing salt precipitation across steps, the telescoped sequence avoids solids-induced pressure drift and residence time variability, ensuring clog-free operation within pump constraints.

While the ‘reaction modification’ strategy demonstrates effective management of solids in continuous flow systems, the careful selection of bases, solvents, and solvent quantities remains critical to achieving environmentally benign and sustainable operation. Selecting acid-scavenging bases that form low-melting ionic liquids prevents base·HX salt precipitation, stabilises viscosity and pressure drop, and reduces fouling on reactor walls, enabling piston/syringe pump delivery at molar concentrations typical of scale-up. These considerations are closely aligned with the principles of green chemistry and strengthen the scalability and industrial applicability of continuous flow processes, particularly in the pharmaceutical and specialty chemical industries.

### Solids-handling strategies comparative summary

The diverse strategies discussed in this review highlight that no single approach universally addresses the challenges of solid management in continuous flow systems. Each technique, ranging from packed bed immobilisation and dynamic mixing reactors to mechanochemical platforms, Pickering emulsions, and chemical route modifications, offers distinct advantages and limitations shaped by operating windows, solid type, and scalability requirements. This section consolidates these strategies into a comparative framework, enabling rapid evaluation of their applicability, performance benefits, and potential risks. By summarising key parameters such as typical solid scenarios, operating conditions, failure modes, and scale-up considerations, the table below serves as a practical reference for selecting robust, sustainable, and solids-compatible solutions in modern continuous flow manufacturing.

Table 1 provides a side-by-side comparison of these strategies, highlighting their operating windows, advantages, limitations, and scale-up considerations for quick decision-making.

**Table 1:** Solids-handling strategies in continuous flow chemistry comparative summary

Strategy	Typical solid scenario	Operating window (indicative)	Advantages	Typical failure modes/risks	Scale-up notes	Key refs
Packed-bed reactors (fixed-bed; columns; modular ePTFE cartridges)	Immobilized reagents/catalysts; scavengers; salt capture	Pressure-driven flow; broad T range; liquid/gas co-feed	High surface area contact; easy separation; modularity	Channelling; pressure drop; fouling/attrition; uneven RTD	Industrial precedent is strong; diffuser design and bed engineering mitigate maldistribution	^57–62,67,68,72,74,76^
CSTR cascades (photo-CSTR; miniature cascades, Agitated Cell Reactor (ACR))	Slurries, precipitates, insoluble bases; multi-step telescoping	Broad RTD; moderate to long residence times; continuous agitation	Robust solids handling; tolerance to phase heterogeneity; GMP-ready	Sedimentation if agitation is insufficient; broad RTD may affect selectivity; particle attrition at high shear; seal leakage	Scales via numbering-up; vertical orientation reduces clogging	^134–137,145–148^
Spinning Disc Reactors (SDR)	Salt formation in fast organolithium/diazonium chemistry; solid product formation in situ	Thin-film at high shear; short residence (seconds–minutes)	Intense mass/heat transfer; tolerance to some precipitation	Solids adherence to rotor; intermittent pressure fluctuation	Effective for hazardous/fast steps; pair with ACR for longer runs	^139,142,146^
Agitated tubular reactors (ATR)	Viscous slurries; ion-exchange/co-precipitation with solids	Mechanical agitation along tube; controlled RTD	Improved suspension stability; intensified mixing	Mechanical wear; complex hydrodynamics if poorly tuned	Emerging scale-up; validated in intensified separations	^140,141^
Oscillatory flow reactors (OFR/COBR)	Suspension stability for particle-laden streams; controlled RTD; low shear	Oscillation frequency/amplitude tuned; near-plug-flow RTD with baffles/cells	Good solids suspension; low shear mixing; scalable via diameter/length	Bypass/back-mixing if poorly configured; complexity in design	Widely studied; add subsection to strengthen dynamic mixing coverage	^33,150,157,158^
Pickering emulsions (PE)	Solid particles at liquid–liquid interface; triphasic catalysis	Slug/Taylor or droplet microfluidics; energy-free stabilization	Pressure-drop minimal; droplet size control; catalyst recovery	Droplet polydispersity; interfacial fouling if particles aggregate	Amenable to numbering-up; control droplet size at scale	^31,91,126^
Colloidal nanoparticle suspensions; SMBR photocatalysis	Mobile nanocatalyst slurries; gas–liquid–solid triphasic	Nanofluids; serial micro-batch reactors; UV/visible photo	High activity; simple recovery; recyclability demonstrated	Aggregation; light penetration limits; sedimentation	High-speed circulation mitigates settling; positive recyclability data	^98,128–130^
Reaction design modifications (bases/solvents/routes)	Salt by-products (base·HX); insoluble reagents/products; adjusting stoichiometry; changing solvent systems; low concentrations	Tailored solvent/base; ionic liquid-forming scavengers	Prevents precipitation/clogging; enables telescoping	Viscosity increases at high loadings; cost of special bases; large solvent volumes; high environmental impact; Dilution reduces throughput	Demonstrated in multistep API intermediates; Optimise solvent recycling	^25,159,160^
Flow mechanochemistry (TSE/JSSR; shear reactors)	Solid-only or paste feeds; solvent-free or low solvent	Controlled torque/screw config; 2–300 s residence typical	High STY; green metrics; robust to solids	Nozzle fouling (slurries); heat management	Demonstrated kg-scale; integration with telescoped flows	^30,34,86,89,90^

## Outlook

The diverse advancement of continuous flow chemistry critically depends on the development of robust, integrated platforms for solid handling. Achieving this requires the convergence of innovative chemical methodologies, sophisticated reactor architectures, specialised equipment, and state-of-the-art in-line monitoring technologies. This review has highlighted a range of solutions, including reaction design modifications and engineering breakthroughs such as spinning disc reactors, sonicated flow systems, agitated tubular reactors, and packed-bed configurations, all of which have shown effectiveness in mitigating clogging and improving process reliability.

Looking ahead, the next frontier is the development of universally adaptable platforms that not only manage solids with ease but also preserve the seamless continuity, scalability, and transformative advantages that define continuous flow chemistry. Achieving this will require deeper integration of chemistry and engineering principles, alongside creative approaches to reactor design and process intensification. Continued interdisciplinary research in this domain will be essential to fully realise the potential of continuous flow chemistry. As these innovations mature, continuous flow chemistry is poised to transcend its current limitations and establish itself as a cornerstone of modern chemical manufacturing, driving sustainable production in pharmaceutical and specialty chemical sectors.
